# Fine mapping of the major QTL for seed coat color in *Brassica rapa* var. Yellow Sarson by use of NIL populations and transcriptome sequencing for identification of the candidate genes

**DOI:** 10.1371/journal.pone.0209982

**Published:** 2019-02-04

**Authors:** Huiyan Zhao, Urmila Basu, Berisso Kebede, Cunmin Qu, Jiana Li, Habibur Rahman

**Affiliations:** 1 Department of Agricultural, Food and Nutritional Science, University of Alberta, Edmonton, AB, Canada; 2 Chongqing Engineering Research Center for Rapeseed, College of Agronomy and Biotechnology, Southwest University, Beibei, Chongqing, People’s Republic of China; College of Agricultural Sciences, UNITED STATES

## Abstract

Yellow seed is a desirable trait in *Brassica* oilseed crops. The *B*. *rapa* var. Yellow Sarson carry unique yellow seed color genes which are not only important for the development of yellow-seeded oilseed *B*. *rapa* cultivars but this variant can also be used to develop yellow-seeded *B*. *napus*. In this study, we developed near-isogenic lines (NILs) of Yellow Sarson for the major seed coat color QTL SCA9-2 of the chromosome A9 and used the NILs to fine map this QTL region and to identify the candidate genes through linkage mapping and transcriptome sequencing of the developing seeds. From the 18.4 to 22.79 Mb region of SCA9-2, six SSR markers showing 0.63 to 5.65% recombination were developed through linkage analysis and physical mapping. A total of 55 differentially expressed genes (DEGs) were identified in the SCA9-2 region through transcriptome analysis; these included three transcription factors, Bra028039 (NAC), Bra023223 (C2H2 type zinc finger), Bra032362 (TIFY), and several other genes which encode unknown or nucleic acid binding protein; these genes might be the candidates and involved in the regulation of seed coat color in the materials used in this study. Several biosynthetic pathways, including the flavonoid, phenylpropanoid and suberin biosynthetic pathways were significantly enriched through GO and KEGG enrichment analysis of the DEGs. This is the first comprehensive study to understand the yellow seed trait of Yellow Sarson through employing linkage mapping and global transcriptome analysis approaches.

## Introduction

Brassica oilseed crops, such as *Brassica napus* L. (AACC, 2*n* = 38), *Brassica rapa* L. (AA, 2*n* = 20) and *Brassica juncea* L. Czern. & Coss. (AABB, 2*n* = 36), are the important sources of edible oil for human consumption and protein-rich meal for livestock feed. Yellow seed is a desirable trait in these crops due to its association with higher oil and protein, lower fiber and pigments contents; therefore, breeding for yellow-seeded Brassica oilseed crops is an important goal to many breeding programs [1–4]. Natural variants of yellow-seeded type have been found in *B*. *rapa*, *B*. *juncea* and *Brassica carinata* L. (BBCC, 2*n* = 34), but not in *B*. *napus*; however, yellow-seeded *B*. *napus* germplasm have been developed by several researchers mainly through interspecific hybridization involving the above-mentioned yellow-seeded variants (for review, see [5; 6–10]), as well as through utilizing the yellow seed color genes of *B*. *rapa* alone [11, 12].

Seed color in Brassica results from deposition of pigments, especially the oxidized proanthocyanidins, which are the products of the secondary metabolic pathways, such as phenylpropanoid and flavonoid biosynthetic pathway (for review, see [13; 14–19]). In *Arabidopsis thaliana* L., dozens of mutants for lack of seed coat pigments, named as *transparent testa* (*tt*) mutants, were developed, and the genes, named as *TT* genes, involved in different stages of flavonoid biosynthetic pathway have been identified [20, 21]. Homologous *TT* genes expressing abnormally in the yellow seeds or involved in the formation of yellow seed trait in Brassica have been reported by several researchers [17, 19, 22–24].

Traditional genetic analysis showed that one to three genes are involved in the control of the formation of brown/black seed coat color while the yellow seed results from homozygous recessive condition at all loci [25–29]. Molecular mapping of seed coat color in *B*. *rapa* by different researchers, such as Lou et al. [30], Kebede et al. [31] and Bagheri et al. [32], using populations derived from crossing of brown-seeded type to Yellow Sarson (*B*. *rapa*), identified a major QTL on the chromosome A9; Kebede et al. [31] also detected a minor QTL (SCA9-1, explained about 4% phenotypic variance) on the same chromosome exhibiting a significant interaction with the major QTL (SCA9-2, explained about 55% phenotypic variance) of A9. Li et al. [17] reported the seed coat color gene *B*. *rapa Transparent Testa 8* (*BrTT8*) on A9 of *B*. *rapa* var. Yellow Sarson and provided evidence that the translucent seed coat color giving the yellow seed trait in Yellow Sarson is due to insertion of a *Helitron* transposon in this gene. Xiao et al. [33] and Wang et al. [23, 34] mapped a major seed coat color gene on A9 by using a yellow-seeded *B*. *rapa* landrace Dahuang and provided evidence for *BrTT1* to be the candidate gene for this trait. Cloning and functional analysis of this gene showed that three missense mutations in *BrTT1* are responsible for the yellow seed trait in Dahuang. Zhang et al. [22] and Ren et al. [35, 36] mapped a major seed coat color locus on A6 (≈R6) of Chinese cabbage (*B*. *rapa*) and provided evidence that a 94-base deletion in *Transparent Testa Glabra1* (*TTG1*) gene resulted the yellow seed color in this variant of *B*. *rapa*. Thus, it is apparent that more than one major locus controlling seed coat color can be found in the *Brassica* A genome, and more than one *TT* gene might be involved in the control of this trait.

With the development of high-throughput transcriptome sequencing, it is possible to capture the whole spectrum of transcripts and reveal their involvement in biological processes of a specific tissue or at specific stage of development of an organism (for review, see [37; 38–40]). The RNA-Seq analysis can also identify novel transcripts, as well as other variations in the genome, such as single nucleotide polymorphism (SNP), insertion and deletion (Indels) and alternative splicing (AS), which can not only provide information of the candidate genes, but can also be used for the development of molecular markers [41–43]. This information can be further utilized to understand the molecular mechanism or the biosynthetic pathways involved in the control of a trait [18, 39, 44]. RNA-Seq can also be combined with molecular mapping for identification of the candidate genes and development of markers for use in marker-assisted breeding [38, 40, 44–47]. In Brassica, transcriptome analysis of the whole seed or its components has been done to understand the genes involved in the biosynthetic pathway and seed coat color formation in *B*. *napus* [44, 48] and *B*. *juncea* [18]. Given that the *B*. *napus* and *B*. *juncea* are amphidiploid species, each carrying two genomes and these genomes are evolved from a common prototype through polyploidization and re-arrangements [49]; this can introduce complexity in research and identification of a gene. Therefore, important knowledge can be gained through working with a simpler genome species, like *B*. *rapa*. For example, Kebede et al. [31] detected a second QTL for seed color on A9, Kebede and Rahman [50] detected a QTL for silique length on A5, and Rahman et al. [51] detected a major QTL for seed glucosinolate content on A2 while working with the diploid species *B*. *rapa*; these QTLs, however, could not be found in literature from studies with the amphidiploid species.

The objective of this study was to fine map the major seed color QTL in *B*. *rapa* by use of a set of near-isogenic lines (NILs) differing for seed color and to identify the candidate genes involved in the control of this trait through transcriptome sequencing of the developing seeds of the NILs. Knowledge gained from this research can be used in marker-assisted selection for the development of yellow-seeded Brassica oilseed crops, as well as for the development of gene-based molecular markers.

## Materials and methods

### Plant materials

Near-isogenic BC_4_S_1_ and BC_4_S_2_ (four backcross followed by one and two time self-pollination) populations were developed from a cross between two self-compatible *B*. *rapa* lines, Sampad (Yellow Sarson, yellow seed) and 3–0026.27 (yellowish brown seed), and using Sampad as the recurrent parent. For this, marker-assisted backcrossing technique was applied where the plants heterozygous for seed color were identified using the co-dominant SCA9-2 QTL markers of A9 reported by Kebede et al. [31]. From this backcross program, the BC_4_ plants heterozygous for seed color were self-pollinated for BC_4_S_1_ seeds; seed color of the BC_4_S_1_ seed families was brown. Fifty five plants of a single BC_4_S_1_ family were grown in a growth chamber (20°C, 16 h light, 8 h dark) and were self-pollinated by bag isolation for BC_4_S_2_ seeds; all these plants were also genotyped with simple sequence repeat (SSR) markers from the two QTL regions, SCA9-1 and SCA9-2, of A9 [31]. Segregation for SSR marker CB10022A from the QTL region of SCA9-2 was found in this population where the plants carrying Sampad allele in homozygous condition produced uniform yellow seeds, while the plants homozygous for 3–0026.27 allele or heterozygous for Sampad and 3–0026.27 alleles produced brown seed (S1 Fig). In contrast, all 55 BC_4_S_1_ plants were homozygous for Sampad allele for the SSR marker CB10103A from the QTL region SCA9-1; this suggests that only the allele of the major QTL SCA9-2 were segregating in this population, as could be expected in a NIL population.

Based on genotypic data of the BC_4_S_1_ plants and phenotypic data of the harvested BC_4_S_2_ seeds, a total of 582 BC_4_S_2_ plants from five yellow-, five homozygous brown- and five heterozygous brown-seeded BC_4_S_2_ families were grown in a greenhouse. All these plants were genotyped with SSR markers and phenotyped of seed color for fine mapping of the seed color gene.

Descriptive statistics of the BC_4_S_1_ and BC_4_S_2_ populations, such as mean and standard error, were calculated in Microsoft Excel 2003, Pearson’s correlation coefficient and one-way analysis of variance (ANOVA) were calculated using the software program Statistical Product and Service Solutions (SPSS) [52].

Seeds of the BC_4_S_1_ plants were analyzed for seed oil and protein contents by Near Infrared (NIR) spectroscopy method and reported as percent of whole seed at 0% moisture basis.

### DNA extraction, marker development, genotyping and fine mapping

Leaf sample from one-month-old BC_4_S_1_ and BC_4_S_2_ plants were collected and genomic DNA was extracted following the method described by Edwards et al. [53]. Forty new SSR markers, designed from the SCA9-2 QTL region and by use of Brassica genome sequence information (http://brassicadb.org/brad/datasets/pub/Genomes/Brassica_rapa/V1.0/V1.5/) [54], were tested on the two parents for polymorphism; the polymorphic markers, as well as the published QTL markers of SCA9-2 [31] were used to genotype the BC_4_S_2_ plants.

Polymerase chain reaction (PCR) was done on a total volume of 15 μL, containing 3 μL Promega 5×GoTaq Flexi Buffer, 1 μL MgCl_2_ solution (25 mM), 0.3 μL dNTPs (10 mM), 0.3 μL forward primer (20 μM), 0.3 μL reverse primer (20 μM), 0.1 μL Promega GoTaq DNA polymerase (5 U/μL), 2 μL template DNA (25 ng/μL) and 8 μL nuclease-free water. The following conditions were set for PCR: initialization was at 94°C for 5 min followed by 40 cycles of amplification, where each cycle consisted of 45 s at 94°C for denaturation, 45 s at the primer melting temperature for annealing, and 1 min at 72°C for elongation. The final elongation was for 10 min at 72°C, and 30 min at 4°C for final hold. PCR products were detected by 1% agarose gel electrophoresis or analyzed by capillary sequencer, 3730 DNA Analyzer (Applied Biosystems, USA). For the capillary sequencing, fluorescent dyes, 6-FAM, VIC, NED or PET (ThermoFisher Scientific, USA) and GeneScan-500 LIZ Size Standard (Applied Biosystems, USA) were included in the PCR reaction. The chromatograms were analyzed using Genemapper software v3.0 (Applied Biosystems, USA).

### RNA extraction and cDNA library construction

Self-pollinated seeds at the age of 10, 20, 30, 40 and 50 days after pollination (DAP) were collected from all above-mentioned 55 BC_4_S_1_ plants. However, after confirmation of their genotype through SSR marker analysis of the BC_4_S_1_ and BC_4_S_2_ plants, and progeny test result of the BC_4_S_1_ plants (i.e. seed color of the BC_4_S_2_ plants), developing seeds from four yellow-seeded and four homozygous brown-seeded BC_4_S_1_ plants were selected and mixed to constitute the yellow- and brown-seeded bulks for RNA-seq analysis. Thus, the total number of bulks was 2 seed color type × 5 growth stages × 2 replicates = 20. RNA extraction was done using a RNeasy mini kit (QIAGEN, Canada), and the first-strand cDNA synthesis was done using a SuperScript II Reverse Transcriptase kit (Invitrogen, USA).

### Transcriptome sequencing and differentially expressed genes (DEGs) calling

Twelve of the above-mentioned 20 bulk RNA samples of the BC_4_S_1_ plants, collected at 20, 30 and 40 DAP, were used for RNA sequencing. Preparation of the cDNA libraries and sequencing (paired-end 150 bp) was done by Novogene (https://en.novogene.com/) using the Illumina Hiseq platform. After quality control of the raw reads (Q20 score ≥ 95%, Q30 score ≥ 88%), the clean reads were mapped to the *B*. *rapa* reference genome (ftp://ftp.ensemblGenomes.org/pub/plants/release-37/fasta/brassica_rapa/dna/) with the gene annotation files (ftp://ftp.ensemblGenomes.org/pub/release-23/plants/gtf/brassica_rapa/) by using the default parameters in TopHat2 v.2.1.1 [55]. Mapping information from all samples was combined and assembled using the Cufflinks assembler to identify the novel genes and optimize the known transcripts [56]. Normalized gene expression level was measured by Fragments Per Kilobase of transcript sequence per Millions base pairs sequenced (FPKM) method [57] using the HTSeq software. DESeq software [58, 59] was used to screen the DEGs, and the filtered DEGs (q-value<0.05) were used for clustering and Venn Diagram analysis. The sequence data was submitted to the Sequence Read Archive (SRA) database in NCBI (Accession number: PRJNA512955).

### Quantitative real-time PCR analysis (qRT-PCR) of selected genes

Based on RNA-seq data analysis and the target gene mapping result, 13 genes from the SCA9-2 QTL region and 19 genes from different biosynthetic pathways, such as phenylpropanoid, flavonoid, lignin and fatty acid biosynthetic pathways, were selected for further validation by qRT-PCR method. All 20 bulks of RNA of the yellow- and brown-seeded plants, collected at five different stages after pollination (10, 20, 30, 40 and 50 DAP), were used for this analysis. The total reaction volume was 20 μL comprising of 10 μL 2× Promega GoTaq qPCR Master Mix, 1 μL 20 μM forward primer, 1 μL 20 μM reverse primer, 100 ng first-strand cDNA, and nuclear-free water was added to make 20 μL. PCR reaction was performed in StepOnePlus Real-Time PCR Systems (Applied Biosystems) with the PCR profile: 2 min at 94°C for hot-start activation followed by 40 cycles of 3 s at 94°C for denaturation, 30 s at 60°C for annealing and extension, and the dissociation curves were generated after the final amplification cycle by increasing the temperature from 60 to 95°C. The 2^-ΔΔCt^ method [60] was adapted to calculate the expression of the selected genes, and *Actin7* was used as internal reference gene.

### Hierarchical cluster (HCL), SNP and Indel, transcription factor analysis of the DEGs, and development of CAPS markers

Heatmap and HCL analysis of the DEGs based on FPKM vales of the yellow and brown seeds were performed using Multi Experiment Viewer (Mev) software v.4.9.0 [61]. Genome Analysis Toolkit (GATK3) was used for SNP and INDEL calling. CAPS (cleaved amplified polymorphic sequence) markers, based on the SNPs identified in this study, were developed from the target region of SCA9-2 of A9 using the software program dCAPS Finder 2.0 (http://helix.wustl.edu/dcaps/dcaps.html) to detect mutated restriction endonuclease cleavage sites. Details of PCR amplification of the markers and restriction site analysis of the parents and segregating BC_1_S_2_ population is described elsewhere [62].

Multiple sequence alignment analysis of the mutant (yellow seed) and reference genes’ protein sequences was performed using the online Clustal Omega software (https://www.ebi.ac.uk/Tools/msa/clustalo/). Transcription factor and Protein Kinase Identifier and Classifier (iTAK) was used to perform transcription factor analysis [63].

### Gene ontology (GO) and kyoto encyclopedia of genes and genomes (KEGG) enrichment, protein-protein interaction (PPI) analysis

Plant GO term enrichment analysis was done by GOseq [64], and the KEGG enrichment analysis was done using KOBAS 2.0 software [65] and *A*. *thaliana* as the reference species. The PPI analysis of DEGs was conducted in STRING (search tool for recurring instances of neighbouring genes) database (https://string-db.org/) using *B*. *rapa* as the reference organism [66].

## Results

### Phenotype of the NIL populations

The near-isogenic BC_4_S_1_ plants differing for yellow and brown seed color were compared for different seed quality traits (Table A in S1 File). The yellow seed had significantly greater oil as well as sum of oil and protein contents as compared to the brown seeds. Oil and sum of oil and protein content showed significant negative correlation with seed coat color (Table A in S1 File) confirming the importance of the yellow seed for these two seed quality traits.

**Fig 1:**
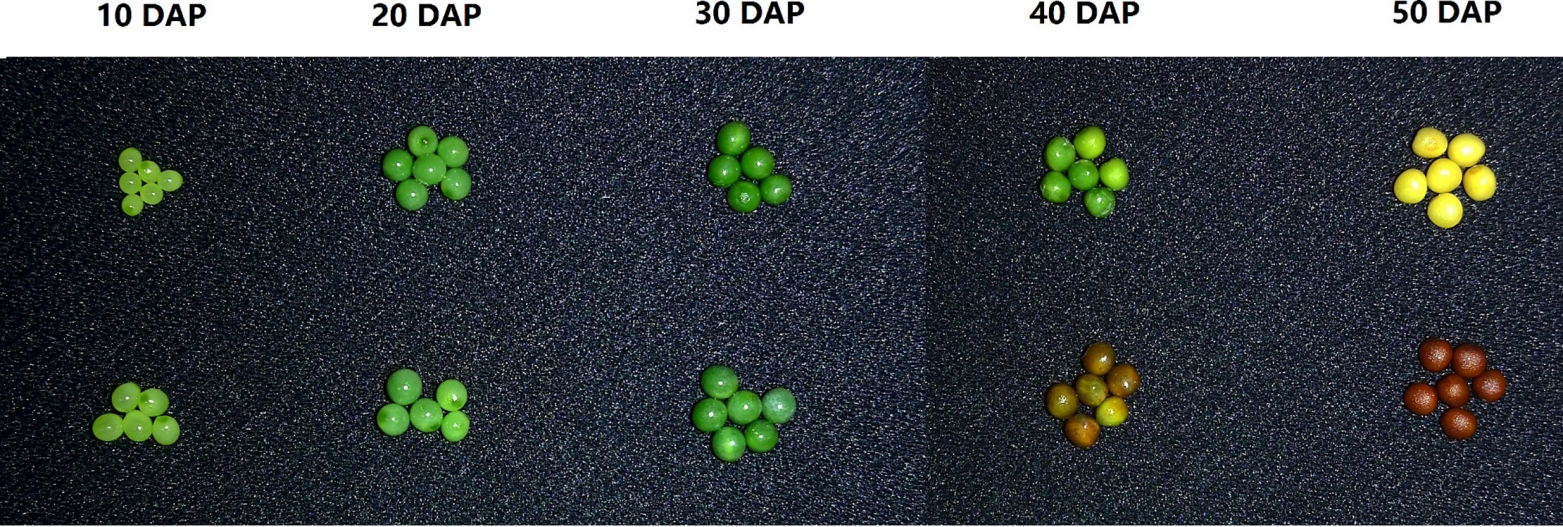
Developing seeds of yellow- and brown-seeded near-isogenic BC_4_S_1_ plants of *Brassica rapa*. DAP = days after pollination.

No seed coat pigment synthesis was observed in the developing seeds up to 30 DAP. At 40 DAP, the brown seeds had significant amount of color, and embryos were still green; and at 50 DAP, the brown seeds turned to completely brown, and the yellow seeds turned to yellow due to the change in embryo color to yellow (Fig 1).

### Fine mapping of the major QTL of A9

**Fig 2:**
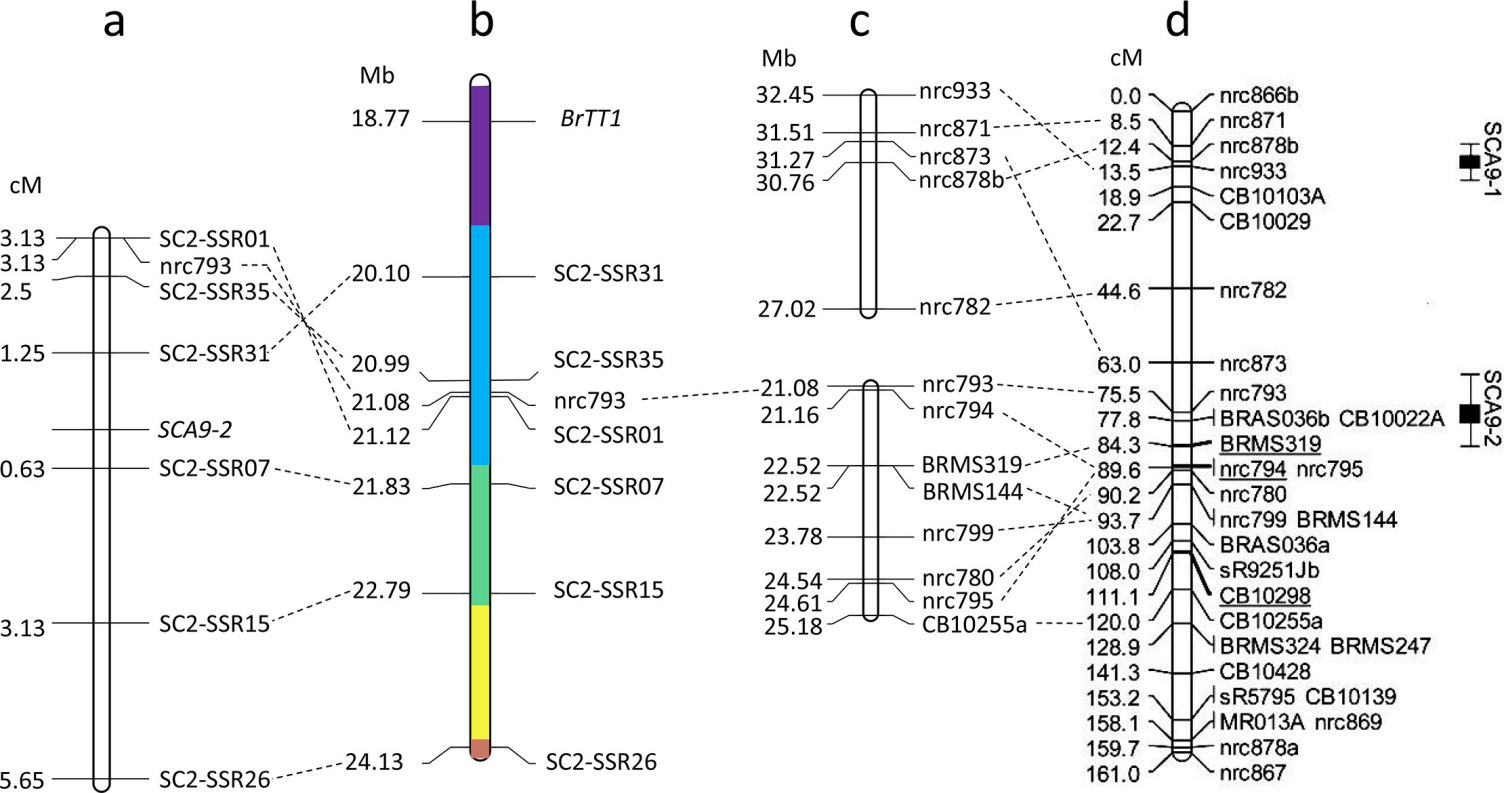
Genetic linkage and physical maps of the chromosome A9 of *Brassica rapa*. (a) A genetic linkage map of a part of A9, carrying the major QTL for seed coat color (SCA9-2), constructed by use of a BC_4_S_2_ population of *B*. *rapa* and genotyping data of six newly-developed SSR markers and one marker used by Kebede *et al*. [31]; the position of the QTL SCA9-2 is shown. Marker position (cM) indicates the distance from SCA9-2. (b) A physical map of the QTL region SCA9-2 of A9 constructed based on one SSR marker used by Kebede *et al*. [31] and the six newly-developed markers co-segregating with seed color; the position of the *BrTT1* gene is also shown. Different colors indicate different scaffolds (purple = scaffold000059, blue = scaffold000040, green = scaffold000081, yellow = scaffold000084, brown = scaffold000045)(c) A physical map of A9 constructed based on sequence information of the markers from the major and minor QTL regions of the linkage group A9 published by Kebede *et al*. [31]; the physical map was constructed through aligning the marker sequences to the *B*. *rapa* reference genome in BRAD database. (d) The genetic linkage map of A9 published by Kebede *et al*. [31]; the major seed coat color QTL SCA9-2 and the minor QTL SCA9-1 are shown.

Based on sequence information of the SSR markers used by Kebede et al. [31] (Fig 2D), a physical map of the chromosome A9 was constructed by aligning the marker sequences to the genome sequence of *B*. *rapa* cv. Chiifu-401 (Fig 2C). Of the seven markers from 0.0 to 44.6 cM region (included the QTL SCA9-1) of this linkage map, four could be positioned on the physical map (27.02 ~ 32.45 Mb) of A9. On the other hand, of the 14 markers from 63.0 to 120.0 cm region (included the QTL SCA9-2), nine could be positioned on the physical map of which eight mapped at 21.08 ~ 25.18 Mb of A9 (Fig 2C); however, the marker CB10022A which showed co-segregation with seed color in the BC_4_S_1_ population could not be positioned on this map.

Based on linkage and physical position of the above-mentioned markers around SCA9-2 (Fig 2C and 2D), a 3.5 Mb region between the markers nrc793 and nrc780 was chosen to increase the density of marker; this region included the marker CB10022A of the genetic map constructed by Kebede et al. [31]. A total of 40 new SSR markers were designed based on *B*. *rapa* genome sequence information, where only six showed polymorphism between the yellow- and brown-seeded plants; these markers positioned at 20.10 to 24.13 Mb in the *B*. *rapa* genome (Table 1; Fig 2B). Of the six new markers, two (SC2-SSR01 and SC2-SSR31) behaved as co-dominant markers while four behaved as dominant marker. The *BrTT1* gene could be positioned at 18.77 Mb position of this map.

**Table 1:** Primer sequence of the simple sequence repeat (SSR) markers, their physical position and genetic distance from the major seed coat color QTL SCA9-2 of A9 of *Brassica rapa*.

Marker	Forward primer	Reverse primer	Expected product size (bp)	Physical position in BRAD database^1^	Physical position in EnsemblPlants database^2^	Genetic distance (cM)
SC2-SSR31	CTTCCATTCCGTTCCACATAA	ACGAGATCGGAAGTGGAGATT	280	A09:20,178,730..20,179,009	A09:20,117,644..20,117,923	1.25
SC2-SSR35	CCACACTTGGTGTTGAAATTATACC	CGGGTCAAGCTCTAGTTTGTAAT	410	A09:20,999,763..21,000,172	A09:20,938,677..20,939,086	2.50
nrc793	TTTCTTGGTGCATCTTTCTTGA	TTGGTATGTAGATTGTTACCTGCTATG	444	A09:21,077,438..21,077,811	A09:21,016,352..21,016,725	3.13
SC2-SSR01	TCACTTTGTTACCTGAACCAC	GAGGTCGAGATCTTTTAGAGG	343	A09:21,117,213..21,117,555	A09:21,056,127..21,056,469	3.13
SC2-SSR07	ATGCGTTTACTTGGTTGTTC	GATGAGAATCTGTACTTTGTGC	316	A09:21,836,870..21,837,185	A09:21,770,784..21,771,099	0.63
SC2-SSR15	GATCCAAAACCAATGGACTA	TGGTTTCACTTTTCTGGTTC	305	A09:22,794,331..22,794,635	A09:22,728,245..22,728,549	3.13
SC2-SSR26	TGCGTCAGAGAGAGAGTTAAA	CAGACAGAACCTGAACTGAAG	306	A09:24,127,579..24,127,884	A09:22,902,883..22,903,188	5.65

^1^*B*. *rapa* chromosome v1.5;

^2^*B*. *rap*a IVFCAASv1

The newly designed six SSR markers and nrc793 were used to genotype the BC_4_S_2_ population derived from five heterozygous BC_4_S_1_ plants to study their linkage association with seed color as well as to construct a genetic linkage map (Fig 2A). Of the seven markers, the co-dominant marker SC2-SSR01 was found to be the closest to nrc793 on the physical map; this marker was capable of distinguishing the yellow-, homozygous brown- and heterozygous brown-seeded plants in the same manner as CB10022A, however, showed 3.13% recombination (3.13 cM away from SCA9-2) in the BC_4_S_2_ segregating population. In contrast, all yellow-seeded BC_4_S_2_ families derived from five yellow-seeded BC_4_S_1_ plants carried the Yellow Sarson allele of SC2-SSR01 in homozygous condition (Table 2). The dominant marker SC2-SSR07 and the co-dominant marker SC2-SSR31 were found to be the most closely-linked flanking markers; these markers are located 0.63 and 1.25 cM away, respectively, from the QTL SCA9-2 (Fig 2A, Table 2). The markers SC2-SSR07 (0.63 cM from SCA9-2, 21.83 Mb), SC2-SSR15 (3.13 cM from SCA9-2, 22.79 Mb) and SC2-SSR26 (5.65 cM from SCA9-2, 24.13 Mb) are located on one side of SCA9-2 and distributed on the Scaffold000081 and Scaffold000045; their location on the genetic linkage map linearly followed the position on the physical map (Fig 2A and 2B). On the other hand, the markers SC2-SSR01 (3.13 cM from SCA9-2, 21.12 Mb), nrc793 (3.13 cM from SCA9-2, 21.08 Mb), SC2-SSR35 (2.5 cM from SCA9-2, 20.99 Mb) and SC2-SSR31 (1.25 cM from SCA9-2, 20.10 Mb) are located on the other side of SCA9-2 and gathered on the Scaffold000040; their order on the genetic (cM) and physical (Mb) was not linear. The gene *BrTT1* was found to be located on the Scaffold000059 (Fig 2B). Based on the results from this study, it can be assumed that the chromosomal region, including the Scaffold000081, Scaffold000040 and Scaffold000059, may carry the candidate gene for seed coat color.

**Table 2:** Segregation for seed color and simple sequence repeat (SSR) marker SC2-SSR01 from the SCA9-2 QTL region of A9 in 15 BC_4_S_2_ families derived from three types of BC_4_S_1_ plants of Yellow Sarson × brown-seeded line 3–0026.027 cross of *Brassica rapa*.

BC_4_S_1_ plant genotype/ phenotype	BC_4_S_2_ families	Total plants	Yellow- seeded plant (#)	Brown- seeded plant (#)	Plants with Yellow Sarson band (#)	Plants with both parents’ band (#)	Plants with 3.0026.27 band (#)
Heterozygous brown seed	2CA.814	32	9	23	9	19	4
	2CA.821	32	8	24	9	14	9
	2CA.831	32	11	21	10	17	5
	2CA.838	32	7	25	7	13	12
	2CA.859	32	5	27	6	18	8
Homozygous yellow seed	2CA.815	32	32	0	32	0	0
	2CA.816	32	32	0	32	0	0
	2CA.843	32	32	0	32	0	0
	2CA.846	29	29	0	29	0	0
	2CA.856	29	29	0	29	0	0
Homozygous brown seed	2CA.827	41	0	41	0	0	41
	2CA.839	80	0	80	0	0	80
	2CA.836	56	0	56	0	0	56
	2CA.848	47	0	47	0	0	47
	2CA.858	44	1	43	1	0	43

### Transcriptome sequencing and reads mapping

A total of 883,392,836 raw reads were obtained from 12 samples which yielded 834,016,880 clean reads with 125.1 billion bases. The clean reads comprised of 431,645,932 reads from the yellow (Y) and 402,370,948 from the brown (B) seed samples. The error rate in analysis was 0.02% for all samples; more than 95% and 88% of the clean reads had the quality score of Q20 and Q30 (percentage of the bases with correct base recognition rate greater than 99.0 to 99.9% of the total bases), respectively, suggesting high quality of this sequencing data. The GC% of the clean bases in different samples varied from 47.3% to 49.2% (Table B in S1 File; S2 Fig). About 53.3% to 57.4% of the clean reads could be mapped on the *B*. *rapa* reference genome (Table B in S1 File) of which about 95.3% to 98.9% were mapped to the exons (Table B in S1 File; S3C Fig). Based on the mapped reads, 34,372 known genes (80.2% of the reference genes) and 1,860 novel genes were detected. The alignment of the number of reads on different chromosomes varied, with the greater number of reads mapped on the longer chromosome (S3A Fig and S3B Fig).

### DEGs between yellow- and brown-seeded samples

A greater number of highly-expressed DEGs were detected in the seeds at 20 and 30 DAP as compared to 40 DAP (S4A Fig). Coefficient of correlation between the two replicates was 0.894 to 0.992 for the RNA-Seq data indicating high reliability and reproducibility of the results from the two biological replicates (S4B Fig). Coefficient of correlation values between the yellow and brown seeds at the same development stage (Y-20 vs. B-20 DAP, Y-30 vs. B-30 DAP and Y-40 vs. B-40 DAP) (*r* = 0.879 to 0.975 with mean of 0.946 ± 0.034) were greater than the values from the different developmental stage (*r* = 0.508 to 0.936 with mean of 0.737 ± 0.131) indicating that the main metabolic processes remained same in both yellow and brown seeds at the same developmental stage, but varied at different developmental stages (S4B Fig).

**Fig 3:**
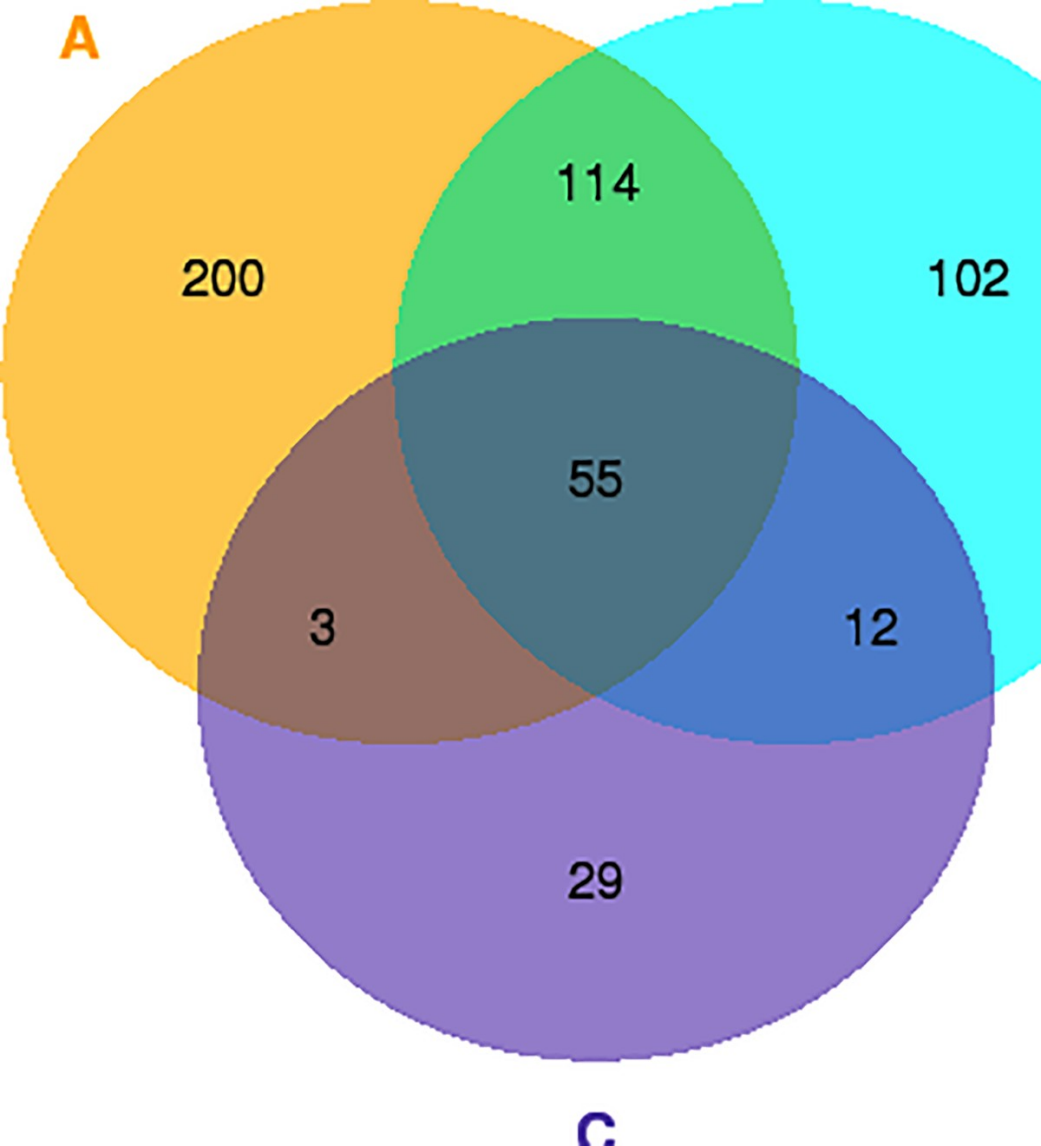
**Venn diagram of the differentially expressed genes (DEGs) in developing seeds of yellow- and brown-seeded near-isogenic BC_4_S_1_ plants of *Brassica rapa* at 20 (a), 30 (b) and 40 (c) days after pollination**.

The number of DEGs showed a decline with the development of seed: 372 DEGs were detected at 20 DAP, 283 at 30 DAP and 99 at 40 DAP (S5 Fig). In total, 515 DEGs were detected between the yellow and brown seeds at these three developmental stages, and these DEGs distributed on 10 chromosomes and 17 scaffolds of *B*. *rapa*. Fifty-five of these DEGs were detected in all three developmental stages (Fig 3, Table C in S1 File). Of the 55 DEGs, 30 (>50%) were mapped to the chromosome A9, providing additional evidence to the existence of the major QTL SCA9-2 on this chromosome. These 30 genes included 5 novel and 25 known genes and located at 8,110,483 to 28,778,767 nt position of A9; 10 of the DEGs, in fact, are located in the SCA9-2 QTL region of 18,744,634 to 22,759,730 nt (Table C in S1 File). In addition to the above-mentioned 30 DEGs, 165 DEGs from A9 also showed significant differences at one or two stages of seed development; 45 of which located in the SCA9-2 region (Table D in S1 File). Thus, a total of 55 (10 + 45) DEGs from the SCA9-2 QTL region constituted the candidate gene pool for seed coat color, and this included 32 down-regulated, 21 up-regulated and two time-varying DEGs (Table E in S1 File).

### Expression profiling analysis of selected genes by qRT-PCR

**Fig 4:**
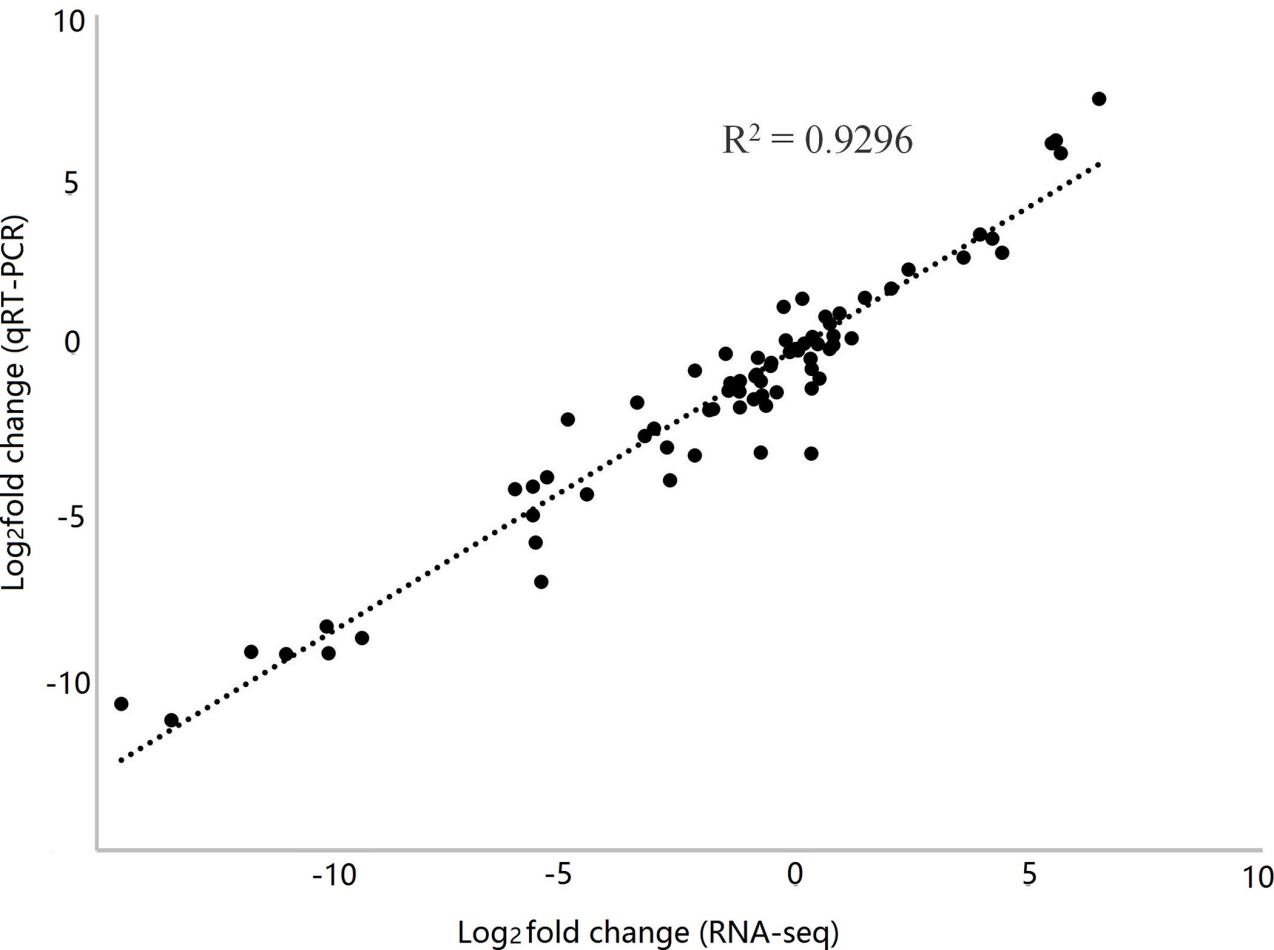
Correlation between the expression values of RNA-seq and qRT-PCR data of the selected 27 genes in yellow and brown seeds of the near-isogenic BC_4_S_1_ plants of *Brassica rapa* at 20, 30 and 40 days after pollination.

**Fig 5:**
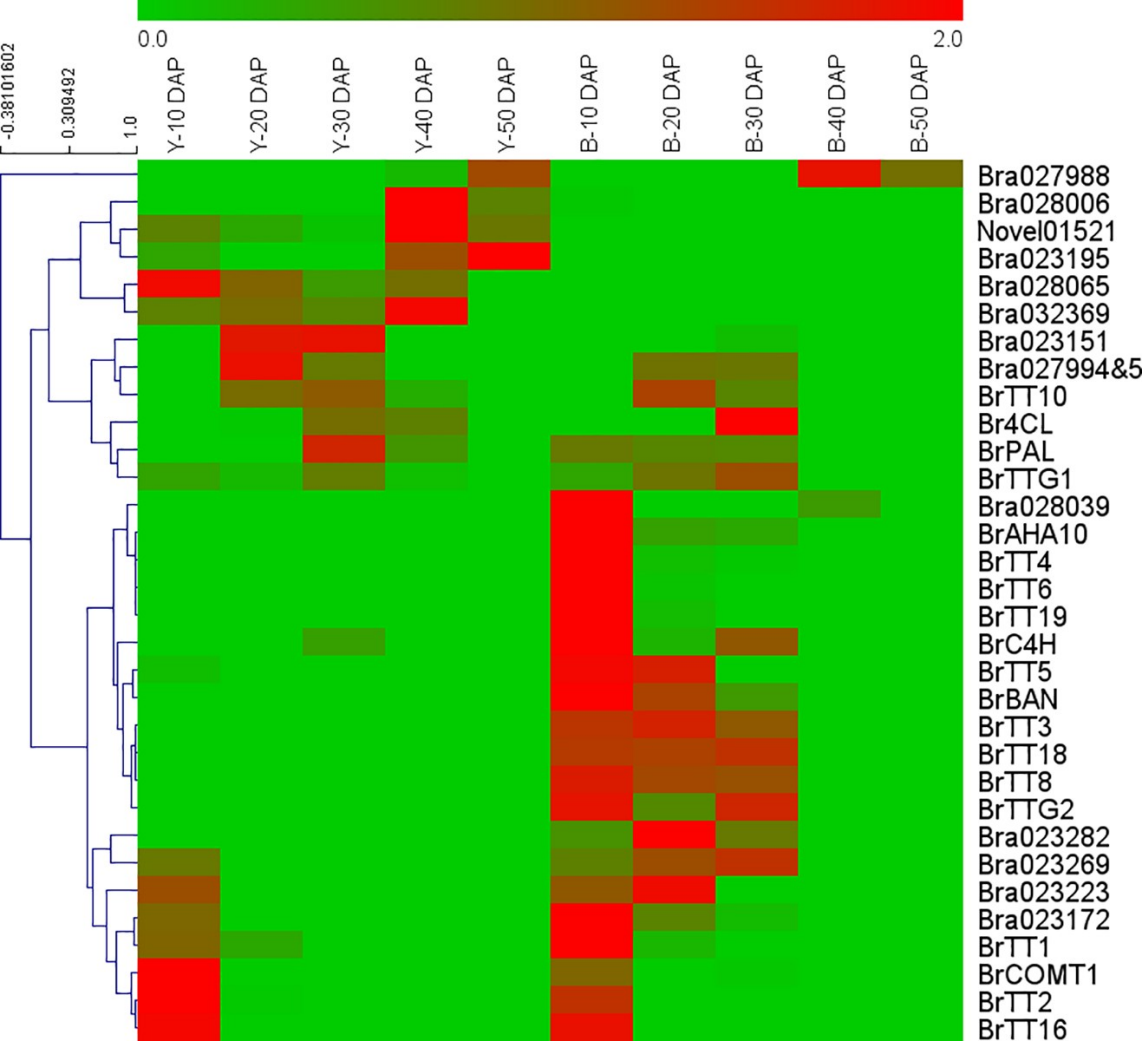
Heatmap of qRT-PCR values of the selected genes from the SCA9-2 QTL region and from the flavonoid biosynthetic pathway in the near-isogenic BC_4_S_1_ yellow (Y) and brown (B) seeds of *Brassica rapa* at 10, 20, 30, 40 and 50 days after pollination (DAP). The red and green colors indicate high and low expression values, respectively.

qRT-PCR analysis was done to study the reliability of the RNA-seq data. For this, a total of 27 single-copy genes, comprising of 12 genes from the SCA9-2 QTL region and 15 genes from the known biosynthetic pathways, were used. Highly significant correlation (R^2^ = 0.94) between the FPKM and 2^-ΔΔCt^ values of these 27 single-copy genes (Fig 4, Table F in S1 File) demonstrated the reliability of the RNA-seq data in our study. Some of the genes used for qRT-PCR analysis, such as *BrTT4*, *BrTT6*, *BrTT19*, *BrAHA10*, *BrTT1*, *BrTT2* and *BrTT16* showed higher level of expression at 10 DAP. Hierarchical cluster analysis of the 19 genes (gene families) from the flavonoid and phenylpropanoid biosynthetic pathways and 13 genes (gene families) from the SCA9-2 QTL region (included 8 up-regulated and 5 down-regulated DEGs, which showed significant fold-change and to be potential candidate genes for seed coat color) grouped these genes into four sub-clusters (Fig 5). Almost all structural genes from the flavonoid biosynthetic pathway and one transcription factor Bra028039 (NAC family protein) were included in one sub-cluster.

### HCL analysis of 515 DEGs

HCL analysis was done to find the genes from the SCA9-2 QTL region co-expressing with the flavonoid pathway genes (might function together with the flavonoid pathway genes) to be considered as the potential candidate genes. Based on the HCL analysis of the FPKM values from the above-mentioned 515 DEGs in yellow and brown seeds, nine sub-clusters were obtained (Pearson’s correlation value = 0.4) (S6 Fig). Nearly all of the DEGs from the flavonoid biosynthetic pathway were included in the sub-cluster 2; this cluster included greater number of DEGs (172 DEGs, 33.4% of all DEGs) as compared to the other sub-clusters and also included 16 DEGs from the major QTL region (SCA9-2) of the chromosome A9 (Table E in S1 File). The expression value of the DEGs from this sub-cluster showed significant differences between yellow and brown seeds at 20 DAP.

### SNP, indel and transcription factor (TF) analysis

SNPs and Indel analysis was done on the above-mentioned 55 (10 + 45) DEGs from the SCA9-2 QTL region where 38 found to carry SNP and one carried both SNP and Indel (Table E in S1 File); these 39 DEGs were considered to be the putative candidate genes. Beside this, seven SNPs were detected in the transcript of *BrTT1* gene (Bra028067). Alignment of the deduced amino acid sequence of the *BrTT1* gene of the yellow-seeded NIL with the reference amino acid sequence in BRAD database and the homologous amino acid sequence of Dahuang (*B*. *rapa*) [34] identified two silent and two sense mutations which were same in both yellow-seeded NIL and Dahuang; however, this analysis also identified three novel sense mutations in the yellow-seeded NIL, i.e. in Yellow Sarson (S7 Fig).

**Fig 6:**
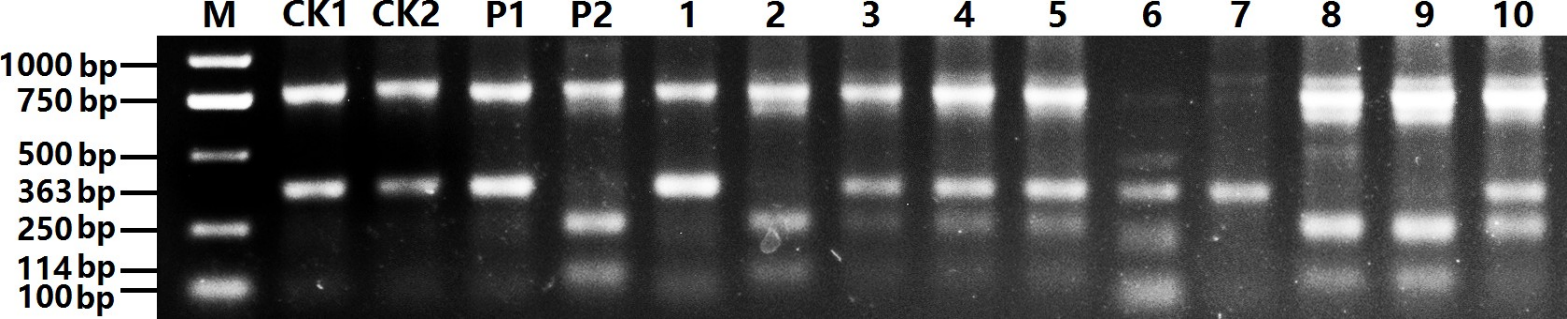
Gel image showing the DNA fragments of the CAPS marker 191BamHI. The lane M is the DNA ladder, and CK1 and CK2 indicate PCR products of Sampad and 3.0026.27 using 191BamHI. P1, P2 and 1–10 indicate digestion results of the PCR products of Sampad, 3–0026.27 and 10 plants of the BC_4_S_2_ population by restriction endonuclease BamHI. Plants 1, 6 and 7 are yellow-seeded, 2, 8 and 9 are homozygous brown-seeded, and 3, 4, 5 and 10 are heterozygous.

A total of seven CAPS markers designed (Table G in S1 File) of which five yielded predicted PCR products; however, restriction endonuclease cleavage sites could not be detected by these markers. The CAPS marker 206TaqI was able to distinguish the yellow and brown-seeded parents based on PCR results; however, it was not able to detect the restriction endonuclease cleavage site. On the other hand, the CAPS marker 191BamHI clearly distinguished the parents and different genotypes of the NIL population based on restriction endonuclease cleavage site results (Fig 6, Table G in S1 File); this further strengthen the reliability of the mapping results reported above (Fig 2).

Transcription factor analysis was done on all the above-mentioned 515 DEGs. A total of 41 TFs were detected among these DEGs; three of these, Bra028039 (NAC family protein), Bra023223 (C2H2 type zinc finger family protein) and Bra032362 (TIFY family protein), were located in the SCA9-2 QTL region (Table E in S1 File).

### GO and KEGG enrichment analysis of the DEGs between the yellow- and brown-seeded samples

GO term enrichment analysis was done to functionally profile (molecular function, biological process and cellular component) the DEGs, and the KEGG enrichment analysis was done to identify the biosynthetic/metabolic pathway in which the DEG’s are involved. GO terms enrichment analysis of the above-mentioned 515 DEGs showed that 15 GO terms were significantly enriched (corrected *p*-value < 0.05) at 20 DAP; this included eleven biological-process terms, three molecular-function terms and one cellular-component term (S8 Fig, Table H in S1 File). Ten of the 11 biological-process terms were found to be related to phenylpropanoid, flavonoid or phenol-containing compound biosynthesis/metabolism (S8 and S9 Figs, Table H in S1 File), while the single term GO:0010345 was found to be involved in suberin biosynthesis. Suberin biosynthesis is known to be related not only to lipid biosynthesis but also to phenylpropanoid biosynthesis as the building blocks of suberin biosynthesis are the products of these two pathways [67]. No significantly enriched GO terms were detected at 30 and 40 DAP (Table H in S1 File).

**Fig 7:**
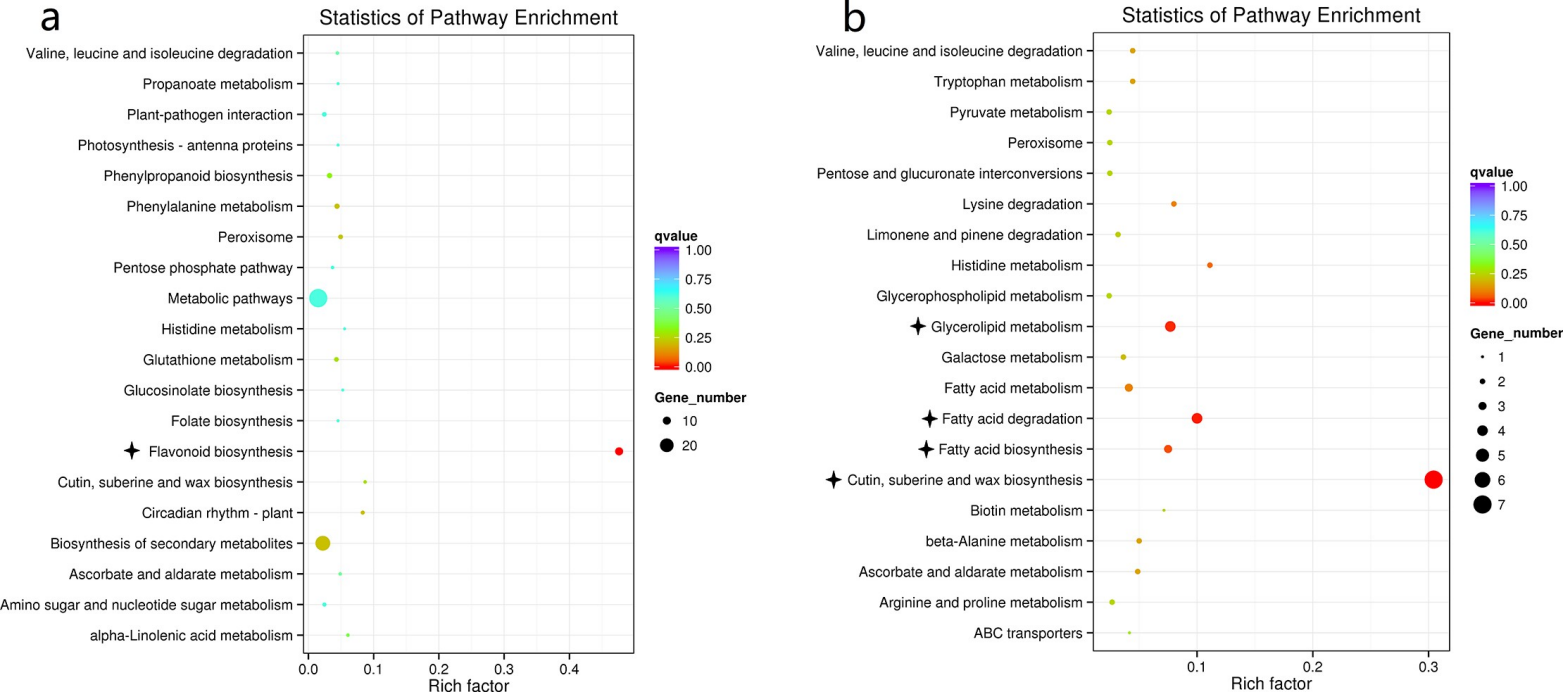
**Scatter plots of enriched Kyoto Encyclopedia of Genes and Genomes pathways based on down- (a) and up- (b) regulated differentially expressed genes (DEGs) in the developing seeds of near-isogenic yellow- and brown-seeded BC_4_S_1_ plants of *Brassica rapa* at 20 days after pollination**. The most significantly enriched 20 pathways are presented. The size of the spherical indicates the number of DEGs. A lower *q*-value (corrected *p*-value) indicates greater enrichment of the pathway. KEGG pathways with *q*-value <0.05 are significantly enriched and labelled with black asterisk. The Rich factor is the ratio of the number of DEGs annotated to the number of all genes annotated in this pathway term. The greater the rich factor, the greater the degree of pathway enrichment.

KEGG pathway enrichment analysis indicated the involvement of flavonoid biosynthetic pathway (ath00941, down-regulated) in all stages of seed development (Table I in S1 File). Other pathways like cutin, suberin and wax biosynthesis (ath00073), fatty acid degradation (ath00071) and fatty acid biosynthesis (ath00061) were found to be up-regulated only at 20 DAP (Table I in S1 File; Fig 7).

Thus, based on the GO and KEGG pathway enrichment analysis, it is evident that phenylpropanoid, flavonoid, suberin and fatty acids biosynthetic pathways are involved in the development of seed coat color (Tables H and I in S1 File), and a total of 139 DEGs were identified based on these analyses (Table J in S1 File).

### PPI analysis of DEGs

**Fig 8:**
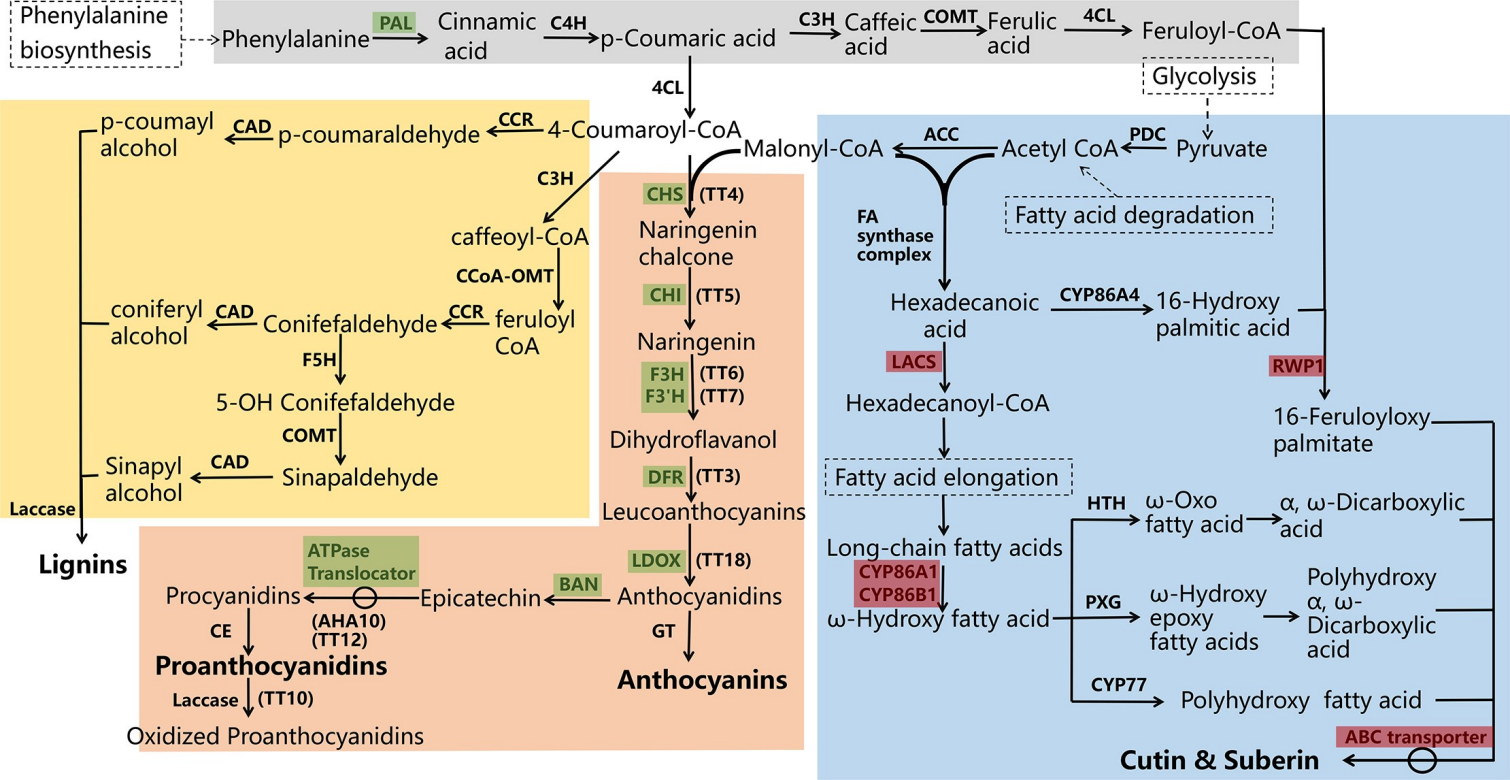
A simplified scheme of phenylpropanoid, lignin, flavonoid and cutin and suberin biosynthetic pathway. These four pathways are shown with four different background colors. The down-regulated genes are marked as green and the up-regulated genes are marked as red. The other related biosynthetic pathways are placed in dotted boxes. PAL = phenylalanine ammonia lyase, C4H = Cinnamate-4-hydroxylase, C3H = Coumarate 3-hydroxylase, 4CL = 4-coumarate:CoA ligase, CCR = Cinnamoyl CoA reductase, CAD = Cinnamyl alcohol dehydrogenase, CCoA-OMT = Caffeoyl CoA O-methyltransferase, F5H = Ferulate 5-hydroxylase, COMT = Caffeic acid/5-hydroxyferulic acid O-methyltransferase, CHS = Chalcone synthase, CHI = Chalcone isomerase, F3H = Flavanone-3-hydroxylase, F3'H = Flavanone-3'-hydroxylase, DFR = Dihydroflavonol 4-reductase, LDOX = Leucoanthocyanidin dioxygenase, BAN = BANYULS, GT = Glucosyltransferase, CE = Condensing enzyme, AHA10 = Autoinhibited H(+)-ATPase isoform 10, TT = Transparent testa, ACC = acetyl-CoA carboxylase, PDC = Pyruvate dehydrogenase kinase, FA = Fatty acids, LACS = Long-chain acyl-CoA synthetase, CYP = Cytochrome P450 family, RWP1 = Reduced levels of wall-bound phenolics 1, HTH = fatty acid omega-hydroxy dehydrogenase, PXG = peroxygenase, ABC = ATP-binding cassette family.

Based on available information of the pathways involved in phenylpropanoid, lignin, flavonoid, and the cutin and suberin biosynthesis [20, 67–69] and the pathway maps available in KEGG database (ath00940: Phenylpropanoid biosynthesis; ath00941: Flavonoid biosynthesis; ath00073: Cutin, suberin and wax biosynthesis; ath00061: Fatty acid biosynthesis; ath00062: Fatty acid elongation; ath00071: Fatty acid degradation) (http://www.genome.jp/kegg/pathway.html), a simplified flow diagram was developed (Fig 8) to understand the involvement of the DEGs identified in this study in the development of seed coat color. The expression and annotation of 242 genes involved in these pathways were collected and summarized in Table K in S1 File.

PPI analysis was performed between the above-mentioned 55 (10 + 45) DEGs from the SCA9-2 QTL region (Table E in S1 File) and the 242 genes (Table K in S1 File) from different enriched and related biosynthetic pathways. Results showed that 41 genes (25 genes from the flavonoid biosynthetic pathway, four genes from the phenylpropanoid biosynthetic pathway, six genes from the suberin biosynthetic pathway, three genes from fatty acid degradation pathway, two genes from fatty acid biosynthetic pathway and one gene from phenoproponoid biosynthetic pathway) interacted with seven DEGs from the SCA9-2 QTL region (S10 Fig). Six of these DEGs (Bra028065, Bra027990, Bra028006, Bra027988, Bra023223, Bra032367) interacted directly or indirectly with the *BrTT1* (Bra028067), *BrTT2* (Bra035532), *BrTT16* (Bra026507, Bra013028) and *BrTT19* (Bra008570, Bra023602) from the flavonoid biosynthetic pathway and one DEG (Bra032295) interacted with BrKAT5 (Bra020673) from the fatty acid degradation pathway (S10 Fig).

Thus, based on annotation of the function of the genes, TF analysis, SNP and Indel analysis, PPI analysis, GO and KEGG enrichment and HCL analysis, a total of 11 DEGs (Table 3, Table E in S1 File) from the SCA9-2 QTL region were identified to be the putative candidate genes involved controlling seed coat color in Yellow Sarson.

**Table 3:** Potential candidate genes for seed coat color identified in the major QTL region SCA9-2 of the chromosome A9 of *Brassica rapa*.

Gene ID	Location	Expression in yellow seeds	Transcription factor	Mutation	PPI with biosynthesis pathway genes	Co-expression with flavonoid pathway genes	KEGG annotation	Gene description
Bra028039	19133850..19135998	Down-regulated	NAC	SNPs and Indel			NAC017; NAC domain containing protein 17	AT1G34190 (E = 2e-223) Arabidopsis NAC domain containing protein 17
Bra023223	20520547..20523792	Down-regulated	C2H2 zinc finger	SNPs	TT16 & TT2	Yes	C2H2-like zinc finger protein	AT1G14580 (E = 2e-093) | zinc finger (C2H2 type) family protein
Bra032362	22159080..22159661	Down-regulated	TIFY	SNPs		Yes	JAZ8; protein TIFY 5A	AT1G30135 (E = 1e-041) TIFY5A | JAZ8 (JASMONATE-ZIM-DOMAIN PROTEIN 8)
Bra027988	19673577..19674003	Down-regulated		SNPs	TT1		transmembrane protein 14C	AT1G33265 (E = 2e-019) | unknown protein
Bra028059	18892053..18893675	Down-regulated		SNPs			essential protein Yae1,N-terminal- domain-containing protein	AT1G34570 (E = 2e-061) | unknown protein
Bra023269	20058621..20058866	Down-regulated		SNPs		Yes	hypothetical protein	AT1G32460 (E = 1e-017) | unknown protein
Bra023172	21023240..21026659	Down-regulated		SNPs		Yes	AGO2; argonaute 2; translation initiation factor 2C	AT1G31280 (E = 0.0) AGO2 | AGO2 (argonaute 2); nucleic acid binding
Bra023130	21507995..21510828	Down-regulated		SNPs			hypothetical protein	AT1G30755 (E = 3e-138) | unknown protein
Bra032370	22098548..22098796	Down-regulated		SNPs			hypothetical protein	AT1G30250 (E = 3e-015) | unknown protein
Bra023188	20887186..20888028	Up-regulated		SNPs			hypothetical protein	AT1G31460 (E = 1e-037) | unknown protein
Bra023133	21471358..21471549	Up-regulated					ribonuclease H-like protein	AT1G10000 (E = 6e-007) | nucleic acid binding / ribonuclease H

### Seed coat color genes/QTL of the A9 chromosome of Brassica

**Fig 9:**
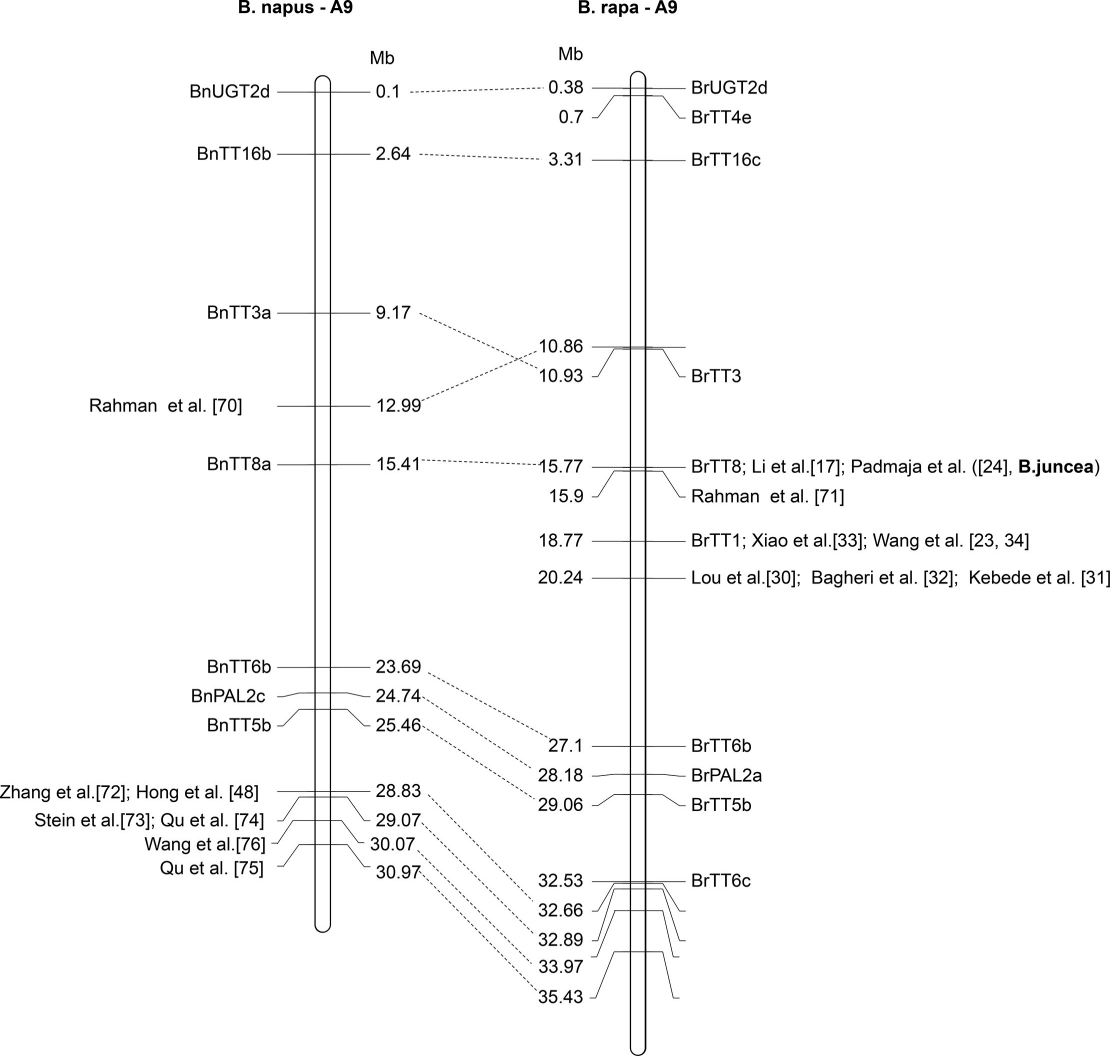
Location of the seed coat color genes or QTLs on the A9 chromosome of *Brassica napus* and *Brassica rapa* based on alignment analysis of the closely linked markers with the reference genome sequences of *B*. *rapa* and *B*. *napus* (http://brassicadb.org/brad/blastPage.php). The physical location of the *transparent testa* (*TT*) genes reported by Qu *et al*. [77] is also included.

To date, several researchers, such as Lou et al. [30], Rahman et al. [70, 71], Zhang et al. [72], Li et al. [17], Xiao et al. [33], Bagheri et al. [32], Padmaja et al. [24], Stein et al. [73], Qu et al. [74, 75], Hong et al. [48], Wang et al.[76] and Wang et al. [23, 34] have reported a major seed coat color gene or QTL on the chromosome A9 of *B*. *rapa*, *B*. *napus* and *B*. *juncea*. By use of the information reported by the above-mentioned researchers, the physical position of the QTL/genes and the markers for seed coat color were aligned to the reference *B*. *napus* and *B*. *rapa* genome (http://brassicadb.org/brad/blastPage.php) and presented in Fig 9. In addition to this, the homologous *TT* genes of A9 of *B*. *napus* and *B*. *rapa* reported by Qu et al. [77] were also included in the Fig 9. Based on this, it was found that the major seed coat color gene or QTL of the chromosome A9 of *B*. *napus* position either on the middle or at one end of the chromosome, while the major QTL of *B*. *rapa* and *B*. *juncea* in all cases position at the middle of this chromosome. The two homologous *TT* genes, *BrTT1* and *BrTT8* (*BjTT8*), which were reported by several researchers [17, 23, 24, 34] to be involved in the control of seed coat color in *B*. *rapa* and *B*. *juncea*, were located at the middle of A9 (Fig 9).

## Discussion

Several studies on seed coat color indicated that a major locus is capable of regulating the formation of seed coat pigments in *B*. *rapa* [17, 22, 28, 33, 35, 78]. In the present study, genetic analysis and molecular mapping of seed coat color by use of NILs further supported this, and confirmed the major QTL SCA9-2 located on the chromosome A9. Kebede et al. [31] also detected a minor QTL SCA9-1 on this chromosome; this QTL shows interaction with the QTL SCA9-2 and has also been found to affect the seed coat color. However, recurrent backcrossing of the F_1_ to the Yellow Sarson parent Sampad with selection for the SCA9-2 QTL alleles resulted in the production of the NILs which carried only the Sampad allele in the SCA9-1, as expected.

SSR markers have been used most commonly due to several advantages, such as high abundance and occurrence throughout the genome, codominant nature, and can be detected through PCR-based assay [79, 80]; therefore, we developed tightly linked SSR markers from the SCA9-2 QTL region by taking the advantage of the *B*. *rapa* genome sequence. Four (SC2-SSR01, nrc793, SC2-SSR35, SC2-SSR31) of the seven linked markers were found to be located at one side of the QTL region (20.10 to 21.12 Mb) of the Scaffold000040. Based on linkage association of these four markers and their physical position in the reference *B*. *rapa* genome, it is apparent that a chromosome inversion might have occurred in the Scaffold000040 of the NILs used in our study. The Yellow Sarson *B*. *rapa* used in this study is genetically distinct from the other variants of this species including the Chinese cabbage [80], therefore, slight structural variation between the chromosomes of Yellow Sarson and the reference genome of Chinese cabbage cv. Chiifu was not unexpected. Cytologically, one of the chromosomes of Yellow Sarson was found to be slightly different from the chromosome of the other oilseed type *B*. *rapa* at pachytene stage [81]. Another possibility could be an error during genome assembly of the Scaffold000040 of A9; however, more markers need to be developed from this genome region to confirm this.

*Transparent testa 1* (*TT1*) and *Transparent testa 8* (*TT8*) are known to be the two important regulatory factors involved in the flavonoid biosynthetic pathway (for review, see [68]). The *TT1* encodes a C2H2 zinc finger protein, which interacts with R2R3-MYB factors and is also known to regulate the expression of several key structural genes, such as *TT4*, *TT3*, *TT18*, *BAN*, and *TT12*, involved in the flavonoid biosynthetic pathway [82, 83]; while the *TT8* encodes a Basic Helix-Loop-Helix (bHLH) protein, can form a ternary complex with *TT2* and *TTG1* and regulates the expression of *BAN* gene; the *TT8* is also under the control of other regulators [84–87]. According to Wang et al. [23, 34], the *BrTT1* is involved in the control of seed coat color in the *B*. *rapa* landrace Dahuang. However, we were not able to detect the *BrTT1* in the SCA9-2 QTL region using FPKM value through RNA-Seq analysis, but qRT-PCR analysis showed that the expression of *BrTT1* was significantly down-regulated at 10 and 30 DAP, not at 20 DAP, in the Yellow Sarson used in this study (S11 Fig). Wang et al. [34] reported significant difference in the level of expression of this gene from 7 to 42 DAP in Dahuang. When comparing the expression level of the other genes from the flavonoid pathway, we found that *BrTT4*, *BrTT5*, *BrTT6*, *BrTT3*, *BrTT18*, *BrBAN*, *BrTT8* and *BrTTG2* were strongly inhibited while a certain level of expression was found for *BrTTG1*, *BrTT2* and *BrTT16* in the yellow seeds; similar level of expression of these genes was also found in Dahuang. However, the pattern of expression of most of the *BrTT* genes was different in these two studied materials ― the highest level of expression was observed in our study at the early stage of seed development (10 and 20 DAP) (Fig 5, S6 and S11 Figs), while the highest level of expression was found at the middle stage (35 DAP) in Dahuang [23]. Lian et al. [88] also provided evidence that *TT1* gene is involved in the control of seed color in *B*. *napus*. Based on these evidences, it is apparent that the mechanism of the regulation of seed coat color in Yellow Sarson is different from the mechanism involved in Dahuang. However, PPI analysis provided evidence for the association of several down-regulated genes from the SCA9-2 QTL region with *BrTT1* (S10 Fig). Thus, based on qRT-PCR analysis and previous reports [23, 34], it appears that *BrTT1* might be involved in the regulation of seed coat color in Yellow Sarson ― whether this gene plays a critical role in this would need further functional investigation.

RNA-Seq analysis as well as qRT-PCR results showed that another important gene *BrTT8* was significantly down-regulated in the yellow seeds at all stages of seed development (S11 Fig), however, this gene could not be detected in the SCA9-2 QTL region. Therefore, *BrTT8* might not be a plausible candidate and this gene might be regulated by other upstream regulatory genes.

In this study, through RNA-Seq and qRT-PCR analysis, we have identified several DEGs in the SCA9-2 QTL region which have not been reported previously; it is highly likely that some of these genes play a major role in the control of seed color. The seed color of the *B*. *rapa* variant Yellow Sarson used in this study is bright yellow and is very unique. Seeds of this variant also lack pigment in the hilum which often can be found in other yellow-seeded lines, and the genetic control of hilum color is independent of the genetic control of seed color [89]. Therefore, it is not unlikely that some other candidate genes are involved in the control of seed color in Yellow Sarson.

RNA-Seq and qRT-PCR analysis also showed that, nearly all of the important structural genes (*BrPAL*, *BrC4H*, *BrTT4*, *BrTT5*, *BrTT6*, *BrTT7*, *BrTT3*, *BrTT18*, *BrBAN*, *BrTT12*, *BrTT19*, *BrAHA10*) and three important TFs (*BrTT8*, *BrTTG2*, *BrTT1*) from the phenylpropanoid and flavonoid biosynthetic pathways were determined to be down-regulated in the yellow seeds (Table K in S1 File; S11 Fig); therefore, it is possible that the candidate gene involved in the regulation of seed coat color in our materials might be an upstream regulatory gene. Indeed, the 55 DEGs detected in the SCA9-2 QTL region included three TFs, Bra028039 (NAC), Bra023223 (C2H2 type zinc finger) and Bra032362 (TIFY); all these contained SNPs or Indel and were down-regulated in the yellow seed, and Bra023223 and Bra032362 clustered with the *BrTT* genes (Table E in S1 File, Table 3). Furthermore, Bra023223 encodes the same type of protein as *BrTT1* (C2H2 zinc finger protein) and was found to interact with the *BrTT2* and *BrTT16* (S10 Fig), therefore, this could be an important candidate gene for seed coat color in our material. Some of the other genes, such as Bra032367 and Bra032295 which showed interaction with the important genes from the flavonoid and fatty acid biosynthesis and degradation pathways, were down-regulated in the yellow seeds; however, none of these genes seems to have a regulatory function based on KEGG annotation and from the description of the homologous genes in *A*. *thaliana*. Several DEGs that encode unknown or nucleic acid binding proteins [90–93], such as Bra023269 (unknown protein) and Bra023172 (argonaute 2, nucleic acid binding protein), etc. (Table 3), which we identified through RNA-Seq analysis, could also be potential candidate genes for seed coat color formation. Therefore, further bioinformatics and functional analysis of the putative candidate genes, would be needed to conclude the candidate gene involved in the blockage of seed coat color formation in Yellow Sarson.

Comparative transcriptome analysis of yellow and brown seeds (seed coat) has been done in *B*. *napus* by Wang et al. [44] and Hong et al. [48] and in *B*. *juncea* by Liu et al. [18]. According to Wang et al. [44], the yellow seed mutant trait in *B*. *napus* is controlled by a single Mendelian gene; transcriptome analysis of the developing seeds of brown- and yellow-seeded NILs showed no significant difference in the level of expression of the *TT* gene orthologues between these two types of seeds; KEGG pathway enrichment analysis by this research group showed that the phenylpropanoid biosynthetic pathway could be enriched. Working with a different set of yellow- and brown-seeded *B*. *napus* NILs, Hong et al. [48] identified few novel genes to be responsible for the formation of seed coat color; however, in this case, expression of the *TT gene* orthologues, including all structural genes (except *BnTT7*) and important regulatory genes (*BnTT8*, *BnTT1* and *BnTT16*) from the flavonoid biosynthetic pathway, were significantly down-regulated in the yellow seeds as compared to the brown seeds. In addition to this, the level of expression of most of these genes was found to reach at the peak at 28, 35 or 42 DAP with the greatest expression at 28 DAP; and the genes (*BnPAL1*, *BnPAL2*, *BnC4H*, *Bn4CL1*, *Bn4CL3*) from the phenylpropanoid biosynthetic pathway were found to be down-regulated in the yellow seeds. In our study, GO and KEGG analysis showed enrichment for the flavonoid (down-regulated), phenylpropanoid (down-regulated), cutin, suberin and wax (up-regulated) biosynthetic pathways and the ABC transporters (up-regulated), as has been reported in *B*. *napus* [48]. However, the other significantly enriched pathways, such as fatty acid degradation (up-regulated) and glycerolipid metabolism (up-regulated), which we identified in this study has not been reported by Hong et al. [48]; in contrast, they reported significant enrichment of plant-pathogen interaction (up-regulated) and plant hormone signal transduction (up-regulated). As found in our study, Hong et al. [48] also reported that the lignin biosynthetic pathway was not enriched in *B*. *napus*. In the case of *B*. *juncea*, transcriptome analysis of yellow- and brown-seeded NILs showed that genes from the flavonoid biosynthetic pathway, such as *BjTT3*, *BjTT18* and *BjBAN*, as well as the genes involved in the phenylpropanoid, phenylalanine, tyrosine and tryptophan biosynthetic pathways are involved in the formation of seed coat color in this species [18].

Thus, it is apparent that, the genes involved in the phenylpropanoid and flavonoid biosynthetic pathways are generally involved in the formation of seed coat color in Brassica. However, some other enriched pathways as well as the expression profile of the flavonoid pathway genes could be different in different materials; this might result from the difference in the major seed coat color genes in these materials. Additionally, the cutin, suberin and wax biosynthetic pathway were also enriched and the genes from this pathway were up-regulated in the Yellow Sarson NILs used in this study, as has also been reported by Hong et al. [48] in *B*. *napus* NILs. Similarly, the tryptophan biosynthetic pathway was found to be enriched in our study as has been reported by Liu et al. [18] in *B*. *juncea* NILs; these pathways are known to have relation with lipid and protein biosynthesis. Thus, our findings provide valuable information on the genetic control and the complex transcriptome dynamics involved in the modulation of seed coat color.

## Supporting information

S1 Fig Capillary electrophorograms and phenotype of the seeds of the near-isogenic BC_4_S_1_ plants. (TIF)

S2 Fig Error rates (a) and GC content (b) distribution of the transcriptome sequencing reads in yellow seed sample (Y1) of BC_4_S_1_ at 20 DAP.
(TIF)

S3 Fig Distribution of the mapped transcriptome reads in 10 chromosomes and to genome regions in yellow seed sample (Y1) of BC_4_S_1_ at 20 DAP. (TIF)

S4 Fig FPKM Distribution of the DEGs at different samples and heat map of the Pearson correlation coefficient between RNA-seq data of the different samples. (TIF)

S5 Fig Volcano Plot of DEGs detected between the near-isogenic BC_4_S_1_ yellow (Y) and brown (B) seed samples at 20, 30 and 40 days after pollination. (padj value>1.3).
(TIF)

S6 Fig Heatmap and of HCL analysis of the expression values of 515 DEGs detected by RNA-seq. The sub-cluster 2 is enlarged; Fb = flavonoid biosynthesis pathway, Sb = subrine biosynthesis pathway. The DEGs from the major QTL SCA9-2 region of A9 chromosome are labelled with blue asterisks.
(TIF)

S7 Fig Multiple sequence alignment analysis using the amino acid sequences of *BrTT1* gene of the yellow-seeded near-isogenic line used in this study (BrTT1-Yellow-RNA-seq), Dahuang (*Brassica rapa*, Wang *et al*. 2016b, BrTT1-Yellow-Wang) and the reference sequence of *Brassica rapa* (BrTT1-Reference). The two silent mutations, two sense mutations which were common in our materials and in Dahuang, one sense mutation found only in Dahuang, and three novel sense mutations found in our materials are marked with blank, blue, yellow and red asterisks, respectively.
(TIF)

S8 Fig The GO enrichment bar graph of the number of DEGs enriched in biological process, cellular component and molecular function. “*”indicates significantly enriched term.
(TIF)

S9 Fig A directed acyclic graph based on the biological process of GO enrichment analysis by the DEGs at 20 DAP. The top eight enriched GO terms (corrected *p*-value <0.05) are shown in square box and the related GO terms are shown in circles. The enrichment degree is illustrated by color shades where the red and yellow shades indicate the higher and lower enrichment degrees respectively. GO term’s name, description, corrected *p*-value, and the number of DEGs/background genes is listed in the boxes.
(TIF)

S10 Fig PPI analysis between the DEGs from the major seed coat color QTL SCA09-2 region and the genes from related biosynthetic pathways. The names of the genes are given in brackets except for the seven genes from the SCA9-2 QTL region. Interaction of the DEGs from the QTL region and the different pathway genes are indicated by red arrows. Edge confidence: The thickness of the line indicates the strength of the supporting data, including neighborhood on chromosome, gene fusion, phylogenetic co-occurrence, homology, co-expression, experimentally determined interaction, database annotated, automated text mining, etc.
(TIF)

S11 Fig qRT-PCR analysis of 13 genes (first 13 in the figure) from the SCA9-2 QTL region and 19 known genes from the flavonoid and phenylpropanoid biosynthetic pathways. “*” = significant difference at 0.05 level (two-tailed); “**” = significant difference at 0.01 level (two-tailed).
(TIF)

S1 File (A) Seed quality traits, (B) sequencing data and mapping results, (C) 55 differentially expressed genes (DEGs) in all seed development stages, (D) 165 DEGs from A9 that differentially expressed in one or two seed developmental stages, (E) 55 DEGs located in the major QTL region of A9, (F) primer sequences for qRT-PCR analysis, (G) CAPS markers developed based on SNPs (H) GO enrichment analysis of the DEGs, (I) KEGG enrichment analysis of the DEGs, (J) 139 DEGs involved in the enriched GO terms and KEGG, and (K) expression and description of the genes from different biosynthetic/metabolic pathways of the near-isogenic *Brassica rapa* plants.
(XLS)

S2 File Supporting information. (XLS)

## References

[1] Stringam GR, McGregor DI, and Pawlowski SH. Chemical and morphological characteristic associated with seed coat colour in rape seed. In: Proc. 4th Int. Rapeseed Conference, Giessen, June 4–8, 1974; p 99–108.

[2] Jönsson R. Breeding for improved oil and meal quality in rape (*Brassica napus* L.)and turnip rape (*Brassica campestris* L.). Hereditas. 1978; 87: 205–218. 10.1111/j.1601-5223.1978.tb01264.x

[3] Rahman MH, Joersbo M, Poulsen MH. Development of yellow‐seeded *Brassica napus* of double low quality. Plant Breed.2008; 120:473–478. 10.1046/j.1439-0523.2001.00639.x.

[4] Jiang J, Wang Y, Xie T, Rong H, Li A, Fang Y, et al Metabolic characteristics in meal of black rapeseed and yellow-seeded progeny of Brassica napus–Sinapis alba hybrids. Molecules. 2015; 20: 21204–21213. 10.3390/molecules201219761. . 26633322 PMC6332043

[5] Chen BY, Heneen WK. Resynthesized *Brassica napus* L.: a review of its potential in breeding and genetic analysis. Hereditas. 1990; 111: 255–263.

[6] Rashid A, Rakow G, Downey RK. Development of yellow seeded *Brassica napus* through interspecific crosses. Plant Breed. 1994; 112:127–134. 10.1111/j.1439-0523.1994.tb00660.x.

[7] Tang Z, Li J, Zhang X, Chen L, Wang R. Genetic variation of yellow-seeded rapeseed lines (*Brassica napus* L.) from different genetic sources. Plant breed. 1997; 116:471–474. 10.1111/j.1439-0523.1997.tb01033.x.

[8] Meng J, Shi S, Gan L. Li Z, Qu X. The production of yellow-seeded *Brassica napus* (AACC) through crossing interspecific hybrids of *B. campestris* (AA) and *B. carinata* (BBCC) with *B. napus*. Euphytica. 1998; 103:329–333. 10.1023/A:1018646223643

[9] Rakow G, Relf-Eckstein J, Raney P. Development of high yielding, disease resistant, yellow-seeded Brassica napus. In Proceedings of the 10th International Rapeseed Congress, Canberra, Australia, 1999; p 26–29.

[10] Li J, Chen L, Wang R, Duan Y. A strategy for breeding of the yellow-seeded hybrid in *Brassica napus* L. In Proceedings of 12th international rapeseed congress, Science Press USA Inc, Genetics and Breeding, 2007; pp 11–13

[11] Rahman MH. Production of yellow-seeded Brassica napus through interspecific crosses. Plant Breed. 2001; 120:463–472. 10.1046/j.1439-0523.2001.00640.x.

[12] Rakow G, Relf-Eckstein J, Olson T.Review and update on the development of yellow seed *Brassica napus* canola. In Proceedings of the 13th International Rapeseed Congress, Prague, Czech Republic, 5–9 June 2011. Abstract, 2011; p55

[13] Winkel-Shirley B. Flavonoid biosynthesis, a colorful model for genetics, biochemistry, cell biology, and biotechnology. Plant Physiol. 2001; 126:485–493. 10.1104/pp.126.2.485. . 11402179 PMC1540115

[14] Debeaujon I, Nesi N, Perez P, Devic M, Grandjean O, Caboche M, et al Proanthocyanidin-accumulating cells in *Arabidopsis* testa: regulation of differentiation and role in seed development. Plant Cell. 2003; 15:2514–2531. 10.1105/tpc.014043. . 14555692 PMC280558

[15] Marles M, Gruber MY. Histochemical characterisation of unextractable seed coat pigments and quantification of extractable lignin in the Brassicaceae. J Sci Food Agric. 2004; 84:251–262. 10.1002/jsfa.1621.

[16] Auger B, Marnet N, Gautier V, Maia-Grondard A, Leprince F, Renard M, et al A detailed survey of seed coat flavonoids in developing seeds of *Brassica napus* L. J Agr Food Chem. 2010; 58:6246–6256. 10.1021/jf903619v . 20429588

[17] Li X, Chen L, Hong M, Zhang Y, Zu F, Wen J, et al A large insertion in bHLH transcription factor *BrTT8* resulting in yellow seed coat in *Brassica rapa*. PloS One. 2012; 7: e44145 10.1371/journal.pone.0044145. 22984469 PMC3439492

[18] Liu X, Lu Y, Yuan Y, Liu S, Guan C, Chen S, et al De novo transcriptome of *Brassica juncea* seed coat and identification of genes for the biosynthesis of flavonoids. Plos One. 2013; 8:e71110 10.1371/journal.pone.0071110. . 23990927 PMC3747200

[19] Qu C, Fu F, Lu K, Zhang K, Wang R, Xu X, et al Differential accumulation of phenolic compounds and expression of related genes in black- and yellow-seeded *Brassica napus*. J Exp Bot. 2013; 64:2885–2898. 10.1093/jxb/ert148. 23698630 PMC3697950

[20] Routaboul JM, Kerhoas L, Debeaujon I, Pourcel L, Caboche M, Einhorn J et al Flavonoid diversity and biosynthesis in seed of *Arabidopsis thaliana*. Planta. 2006; 224(1): 96–107. 10.1007/s00425-005-0197-5. . 16395586

[21] Appelhagen I, Thiedig K, Nordholt N, Schmidt N, Huep G, Sagasser M, et al Update on transparent testa mutants from *Arabidopsis thaliana*: characterisation of new alleles from an isogenic collection. Planta. 2014; 240:955–970. 10.1007/s00425-014-2088-0. 24903359

[22] Zhang J, Lu Y, Yuan Y, Zhang X, Geng J, Chen Y, et al Map-based cloning and characterization of a gene controlling hairiness and seed coat color traits in *Brassica rapa*. Plant Mol Biol. 2009; 69: 553–563. 10.1007/s11103-008-9437-y. . 19039665

[23] Wang Y, Xiao L, Dun X, Liu K, Du D. Characterization of the BrTT1 gene responsible for seed coat color formation in Dahuang (Brassica rapa L. landrace). Mol Breeding. 2017; 37(11): 137 10.1007/s11032-017-0736-3.

[24] Padmaja LK, Agarwal P, Gupta V, Mukhopadhyay A, Sodhi YS, Pental D, et al Natural mutations in two homoeologous TT8 genes control yellow seed coat trait in allotetraploid *Brassica juncea* (AABB). Thero Appl Genet. 2014; 127(2): 339–347. 10.1007/s00122-013-2222-6. 24247234

[25] Mohammad A, Sikka SM, Aziz MA. Inheritance of seed colour in some oleiferous Brassiceae. Indian J Genet Plant Breed. 1942; 2:112–127.

[26] Jönsson R. Yellow-seeded rape and turnip rape. II. Breeding for improved quality of oil and meal in yellow-seeded materials. J Swed Seed Assoc. 1975; 85: 271–275.

[27] Stringam GR. Inheritance of Seed Color in Turnip Rape. Can J Plant Sci. 1980; 60: 331–335. 10.4141/cjps80-054.

[28] Ahmed SU, Zuberi MI. Inheritance of seed coat color in *Brassica campestris* L. variety Toria. Crop Sci.1971; 11:309–310. 10.2135/cropsci1971.0011183X001100020047x.

[29] Rahman MH. Inheritance of petal colour and its independent segregation from seed colour in *Brassica rapa*. Plant Breed. 2001; 120:197–200. 10.1046/j.1439-0523.2001.00607.x.

[30] Lou P, Zhao J, Kim JS, Shen S, Del Carpio DP, Song X, et al Quantitative trait loci for flowering time and morphological traits in multiple populations of *Brassica rapa*. J Exp Bot. 2007; 58(14): 4005–4016. 10.1093/jxb/erm255. . 18048374

[31] Kebede B, Cheema K, Greenshields DL, Li C, Selvaraj G, Rahman H. Construction of genetic linkage map and mapping of QTL for seed color in *Brassica rapa*. Genome. 2012; 55: 813–823. 10.1139/g2012-066. . 23231600

[32] Bagheri H, Pinodelcarpio D, Hahnart C, Bonnema G, Keurentjes J, Aarts MGM. Identification of seed-related QTL in *Brassica rapa*. Span J Agriv Res. 2013; 11(4): 1085 10.5424/sjar/2013114-4160.

[33] Xiao L, Zhao Z, Du D, Yao Y, Xu L, Tang G. Genetic characterization and fine mapping of a yellow-seeded gene in Dahuang (a Brassica rapa landrace). Thero Appl Genet. 2012; 124(5): 903–909. 10.1007/s00122-011-1754-x. . 22120455

[34] Wang Y, Xiao L, Guo S, An F, Du D. Fine Mapping and Whole-Genome Resequencing Identify the Seed Coat Color Gene in *Brassica rapa*. PLoS One. 2016; 11(11): e0166464 10.1371/journal.pone.0166464. . 27829069 PMC5102352

[35] Ren Y, He Q, Ma X, Zhang L. Characteristics of Color Development in Seeds of Brown- and Yellow-Seeded Heading Chinese Cabbage and Molecular Analysis of Brsc, the Candidate Gene Controlling Seed Coat Color. Front Plant Sci. 2017; 8:1410 10.3389/fpls.2017.01410. . 28855913 PMC5558542

[36] Ren Y, Wu J, Zhao J, Hao L, Zhang L. Identification of SSR markers closely linked to the yellow seed coat color gene in heading Chinese cabbage (*Brassica rapa* L. ssp. pekinensis). Biology Open. 2017; 6(2): 278–282. 10.1242/bio.021592. . 28069590 PMC5312096

[37] Wang Z, Gerstein MM. RNA-Seq: a revolutionary tool for transcriptomics. Nat Rev Genet. 2009; 10:57 10.1038/nrg2484. . 19015660 PMC2949280

[38] Pandit A, Rai V, Bal S, Sinha S, Kumar V, Chauhan M, et al Combining QTL mapping and transcriptome profiling of bulked RILs for identification of functional polymorphism for salt tolerance genes in rice (*Oryza sativa* L.). Mol Genet Genomics. 2010; 284(2): 121–136. 10.1007/s00438-010-0551-6. 20602115

[39] Chen J, Tan RK, Guo XJ, Fu ZL, Wang Z, Zhang ZY, et al Transcriptome analysis comparison of lipid biosynthesis in the leaves and developing seeds of *Brassica napus*. Plos One.2015; 10: e0126250 10.1371/journal.pone.0126250. . 25965272 PMC4429122

[40] Qu C, Fu F, Liu M, Zhao H, Liu C, Li J, et al Comparative transcriptome analysis of recessive male sterility (RGMS) in sterile and fertile *Brassica napus* lines. Plos One. 2015; 10:e0144118 10.1371/journal.pone.0144118. . 26656530 PMC4675519

[41] Lalonde E, Ha K, Wang Z, Bemmo A, Kleinman CL, Kwan T, et al RNA sequencing reveals the role of splicing polymorphisms in regulating human gene expression. Genome Res. 2011; 21(4): 545–554. 10.1101/gr.111211.110. . 21173033 PMC3065702

[42] Lopezmaestre H, Brinza L, Marchet C, Kielbassa J, Bastien S, Boutigny M, et al SNP calling from RNA-seq data without a reference genome: identification, quantification, differential analysis and impact on the protein sequence. Nucleic Acids Res. 2016; 44:e148 10.1093/nar/gkw655. . 27458203 PMC5100560

[43] Luo X, Liang X, Liang D, Wang Y, Zhang W, Zhu X, et al Comparative transcriptomics uncovers alternative splicing and molecular marker development in radish (*Raphanus sativus* L.). BMC Genomics. 2017; 18:505 10.1186/s12864-017-3874-4. . 28673249 PMC5496183

[44] Wang F, He J, Shi J, Zheng T, Xu F, Wu G, et al Embryonal control of yellow seed coat locus *ECY1* is related to alanine and phenylalanine metabolism in the seed embryo of *Brassica napus*. G3. 2016; 6:1073–1081. 10.1534/g3.116.027110. . 26896439 PMC4825642

[45] Chen J, Pang W, Chen B, Zhang C, Piao Z. Transcriptome analysis of *Brassica rapa* near-isogenic lines carrying clubroot-resistant and -susceptible alleles in response to *Plasmodiophora brassicae* during early infection. Front Plant Sci. 2016; 6:1183 10.3389/fpls.2015.01183 . 26779217 PMC4700149

[46] Xiong H, Guo H, Xie Y, Zhao L, Gu J, Zhao S, et al RNA-seq analysis reveals pathways and candidate genes associated with salinity tolerance in a spaceflight-induced wheat mutant. Sci Rep. 2017; 7:2731 10.1038/s41598-017-03024-0. PMID: PMC5457441. 28578401 PMC5457441

[47] Huang S, Liu Z, Yao R, Li D, Zhang T, Li X, et al Candidate gene prediction for a petal degeneration mutant, *pdm*, of the Chinese cabbage (*Brassica campestris* ssp. pekinensis) by using fine mapping and transcriptome analysis. Mol Breeding. 2016; 36:26 10.1007/s11032-016-0452-4.

[48] Hong M, Hu K, Tian T, Li X, Chen L, Zhang Y, et al Transcriptomic Analysis of Seed Coats in Yellow-Seeded *Brassica napus* Reveals Novel Genes That Influence Proanthocyanidin Biosynthesis. Front Plant Sci. 2017; 8 10.3389/fpls.2017.01674. . 29051765 PMC5633857

[49] Parkin I. Chasing ghosts: comparative mapping in the Brassicaceae. In Genetics and Genomics of Brassicaceae Plant Genetics and Genomics: Crops and Models, vol 9, Springer, New York, NY, 2011; p153–170

[50] Kebede B, Rahman H. Quantitative trait loci (QTL) mapping of silique length and petal colour in *Brassica rapa*. Plant Breeding. 2015; 133: 609–614. 10.1111/pbr.12193.

[51] Rahman H, Kebede B, Zimmerli C, Yang R. Genetic study and QTL mapping of seed glucosinolate content in *Brassica rapa* L. Crop Sci. 2014; 54:537 10.2135/cropsci2013.06.0391.

[52] George D, Mallery P. SPSS for windows step by step: a simple guide and reference. Computer Software. 2003; 100:357.

[53] Edwards K, Johnstone C, Thompson C. A simple and rapid method for the preparation of plant genomic DNA for PCR analysis. Nucleic Acids Res. 1991; 19:1349 10.1093/nar/19.6.1349. . 2030957 PMC333874

[54] Wang X, Wang H, Wang J, Sun R, Wu J, Liu S, et al The genome of the mesopolyploid crop species *Brassica rapa*. Nat Genet. 2011; 43:1035 10.1038/ng.919. . 21873998

[55] Kim D, Pertea G, Trapnell C, Pimentel H, Kelley R, Salzberg SL. Tophat2: accurate alignment of transcriptomes in the presence of insertions, deletions and gene fusions. Genome Biol. 2013; 14:R36 10.1186/gb-2013-14-4-r36. . 23618408 PMC4053844

[56] Trapnell C, Roberts A, Goff L, Pertea G, Kim D, Kelley DR, et al Differential gene and transcript expression analysis of RNA-seq experiments with TopHat and Cufflinks. Nat Protocols. 2012; 7(3): 562–578. 10.1038/nprot.2012.016. . 22383036 PMC3334321

[57] Mortazavi A, Williams BA, Mccue K, Schaeffer L, Wold B. Mapping and quantifying mammalian transcriptomes by RNA-Seq. Nat Methods. 2008; 5(7): 621–628. 10.1038/nmeth.1226. 18516045 PMC13303166

[58] Anders S, Huber W. Differential expression analysis for sequence count data. Genome Biol. 2010; 11(10): R106 10.1186/gb-2010-11-10-r106. . 20979621 PMC3218662

[59] Anders S, Huber W. Differential expression of RNA-seq data at the gene level—the DESeq package. Embl. 2012.

[60] Schmittgen TD, Livak KJ. Analyzing real-time PCR data by the comparative CT method. Nat Protocols. 3(6): 1101–1108. 10.1038/nprot.2008. 2008; 73 . 18546601

[61] Howe E, Holton K, Nair S, Schlauch D, Sinha R, Quackenbush J. MeV: MultiExperiment Viewer. 2010; 267–277. 10.1007/978-1-4419-5714-6_15.

[62] Neff M, Turk E, Kalishman M. Web-based primer design for single nucleotide polymorphism analysis. Trends Genet, 2002; 18:613–615. 10.1016/S0168-9525(02)02820-2. . 12446140

[63] Zheng Y, Jiao C, Sun H, Rosli HG, Pombo MA, Zhang P, et al iTAK: a program for genome-wide prediction and classification of plant transcription factors, transcriptional regulators, and protein kinases. Mol Plant. 2016; 9:1667–1670. 10.1016/j.molp.2016.09.014. . 27717919

[64] Young M, Wakefield M, Smyth G, Alicia O. Gene ontology analysis for RNA-seq: accounting for selection bias. Genome Biol. 2010; 11: R14 10.1186/gb-2010-11-2-r14. . 20132535 PMC2872874

[65] Xie C, Mao X, Huang J, Ding Y, Wu J, Dong S, et al KOBAS 2.0: a web server for annotation and identification of enriched pathways and diseases. Nucleic Acids Res, 2011; 39:316–322. 10.1093/nar/gkr483. . 21715386 PMC3125809

[66] Szklarczyk D, Morris J, Cook H, Kuhn M, Wyder S, Simonovic M, et al The string database in 2017: quality-controlled protein–protein association networks, made broadly accessible. Nucleic Acids Res, 2017; 45:D362–D368. 10.1093/nar/gkw937. . 27924014 PMC5210637

[67] Franke R, Schreiber L. Suberin—a biopolyester forming apoplastic plant interfaces. Curr. Opin Plant Biol. 2007; 10: 252–259. 10.1016/j.pbi.2007.04.004. . 17434790

[68] Koes R, Verweij W, Quattrocchio F. Flavonoids: a colorful model for the regulation and evolution of biochemical pathways. Trends Plant Sci. 2005; 10: 236–242. 10.1016/j.tplants.2005.03.002. . 15882656

[69] Baud S, Lepiniec L. Physiological and developmental regulation of seed oil production. Progr. Lipid Res. 2010; 49: 235–249. 10.1016/j.plipres.2010.01.001. . 20102727

[70] Rahman M, Li G, Schroeder D, Mcvetty P. Inheritance of seed coat color genes in *Brassica napus* (L.) and tagging the genes using SRAP, SCAR and SNP molecular markers. Mol Breeding. 2010; 26(3): 439–453. 10.1007/s11032-009-9384-6.

[71] Rahman M, Mamidi S, Mcclean P. Quantitative trait loci mapping of seed colour, hairy leaf, seedling anthocyanin, leaf chlorosis and days to flowering in F2 population of *Brassica rapa* L. Plant Breed. 2014; 133:381–389. 10.1111/pbr.12165.

[72] Zhang Y, Li X, Chen W, Yi B, Wen J, Shen J, et al Identification of two major QTL for yellow seed color in two crosses of resynthesized *Brassica napus* line No. 2127–17. Mol Breed. 2011; 28: 335–342. 10.1007/s11032-010-9486-1. .

[73] Stein A, Wittkop B, Liu L, Snowdon R. Dissection of a major QTL for seed colour and fibre content in Brassica napus reveals colocalization with candidate genes for phenylpropanoid biosynthesis and flavonoid deposition. Plant Breed. 2013; 132:382–389. 10.1111/pbr.12073.

[74] Qu C, Hasan M, Lu K, Liu L, Zhang K, Fu F, et al Identification of QTL for seed coat colour and oil content in *Brassica napus* by association mapping using SSR markers. Can J Plant Sci. 2015; 95(2): 387–395. 10.4141/cjps2013-411.

[75] Qu C, Zhao H, Fu F, Zhang K, Yuan J, Liu L, et al Molecular mapping and QTL for expression profiles of flavonoid genes in *Brassica napus*. Front Plant Sci. 2016; 7:1691 10.3389/fpls.2016.01691. . 27881992 PMC5102069

[76] Wang J, Xian X, Xu X, Qu C, Lu K, Li J, et al Genome-wide association mapping of seed coat color in *Brassica napus*. J Agr Food Chem. 2017; 65(26): 5229–5237. 10.1021/acs.jafc.7b01226. . 28650150

[77] Qu C, Zhao H, Fu F, Wang Z, Zhang K, Zhou Y, et al Genome-wide survey of flavonoid biosynthesis genes and gene expression analysis between black- and yellow-seeded *Brassica napus*. Front Plant Sci. 2016; 7:1755 10.3389/fpls.2016.01755. . 27999578 PMC5139615

[78] Hawk JA. 1982 Single gene control of seed color and hypoctyl color in turnip rape. Can J Plant Sci. 62:331–334. 10.4141/cjps82-051.

[79] Piquemal J, Cinquin E, Couton F, Rondeau C, Seignoret E, Doucet I, et al Construction of an oilseed rape (Brassica napus L.) genetic map with SSR markers. Theor Appl Genet. 2005; 111:1514–1523. 10.1007/s00122-005-0080-6. . 16187118

[80] Hobson N, Rahman H, Charles MT. Genome-wide identification of SSR markers in the Brassica A genome and their utility in breeding. Can J Plant Sci. 2016; 96(5): 808–818. 10.1139/cjps-2015-0250.

[81] Röbbelen G. Contributions to the analysis of the Brassica-genome. Chromosoma. 1960; 11:205–228. 10.1007/BF00328652. . 14438262

[82] Sagasser M, Lu G, Hahlbrock K, Weisshaar B. *A*. *thaliana* TRANSPARENT TESTA 1 is involved in seed coat development and defines the WIP subfamily of plant zinc finger proteins. Genes Dev. 2002; 16(1): 138–149. 10.1101/gad.212702. . 11782451 PMC155310

[83] Appelhagen I, Lu GH, Huep G, Schmelzer E, Weisshaar B, Sagasser M. TRANSPARENT TESTA1 interacts with R2R3-MYB factors and affects early and late steps of flavonoid biosynthesis in the endothelium of *Arabidopsis thaliana* seeds. Plant J. 2011; 67(3): 406–419. 10.1111/j.1365-313X.2011.04603.x. . 21477081

[84] Nesi N, Debeaujon I, Jond C, Pelletier G, Caboche M, Lepiniec L. The *TT8* gene encodes a basic helix-loop-helix domain protein required for expression of *DFR* and *BAN* genes in Arabidopsis siliques. Plant Cell. 2000; 12:1863 10.1105/tpc.12.10.1863. . 11041882 PMC149125

[85] Baudry A, Heim MA, Dubreucq B, Caboche M, Weisshaar B, Lepiniec L. *TT2*, *TT8*, and *TTG1* synergistically specify the expression of BANYULS and proanthocyanidin biosynthesis in *Arabidopsis thaliana*. Plant J. 2004; 39(3): 366–380. 10.1111/j.1365-313X.2004.02138.x. 15255866

[86] Baudry A, Caboche M, Lepiniec L. *TT8* controls its own expression in a feedback regulation involving *TTG1* and homologous *MYB* and *bHLH* factors, allowing a strong and cell-specific accumulation of flavonoids in *Arabidopsis thaliana*. Plant J. 2006; 46(5): 768–779. 10.1111/j.1365-313X.2006.02733.x. . 16709193

[87] Xu W, Grain D, Le Gourrierec J, Harscoët E, Berger A, Jauvion V, et al Regulation of flavonoid biosynthesis involves an unexpected complex transcriptional regulation of *TT8* expression, in Arabidopsis. New Phytologist. 2013; 198(1): 59–70. 10.1111/nph.12142. . 23398515

[88] Lian J, Lu X, Yin N, Ma L, Lu J, Liu X, et al Silencing of *BnTT1* family genes affects seed flavonoid biosynthesis and alters seed fatty acid composition in *Brassica napus*. Plant Sci. 2017; 254:32–47. 10.1016/j.plantsci.2016.10.012. . 27964783

[89] Schwetka A. Inheritance of seed colour in turnip rape (*Brassica campestris* L.). Theor Appl Genet. 1982; 62:161–169. 10.1007/BF00293352. . 24270566

[90] Kang J, Guo Y, Chen Y, Li H, Zhang L, Liu H. Upregulation of the AT-hook DNA binding gene *BoMF2* in Ogu-CMS anthers of *Brassica oleracea* suggests that it encodes a transcriptional regulatory factor for anther development. Mol Biol Rep. 2014; 41(4): 2005–2014. 10.1007/s11033-014-3048-2. . 24443226

[91] Lee K, Kang H. Emerging roles of RNA-binding proteins in plant growth, development, and stress responses. Mol Cells. 2016; 39:179–185. 10.14348/molcells.2016.2359. . 26831454 PMC4794599

[92] Park YR, Choi MJ, Park SJ, Kang H. Three zinc-finger RNA-binding proteins in cabbage (*Brassica rapa*) play diverse roles in seed germination and plant growth under normal and abiotic stress conditions. Physiol Plantarum. 2017; 159(1): 93–106. 10.1111/ppl.12488. . 27528428

[93] Luo J, Tang S, Mei F, Peng X, Li J, Li X, et al BnSIP1-1, a trihelix family gene, mediates abiotic stress tolerance and ABA signaling in *Brassica napus*. Front Plant Sci. 2017; 8:e71136 10.3389/fpls.2017.00044. . 28184229 PMC5266734

