# Supplementary figures and images for: Fine mapping of the major QTL for seed coat color in Brassica rapa var. Yellow Sarson by use of NIL populations and transcriptome sequencing for identification of the candidate genes
Source: PLoS One. 2019 Feb 4;14(2):e0209982. doi: 10.1371/journal.pone.0209982 (PMC6361427; doi:10.1371/journal.pone.0209982)

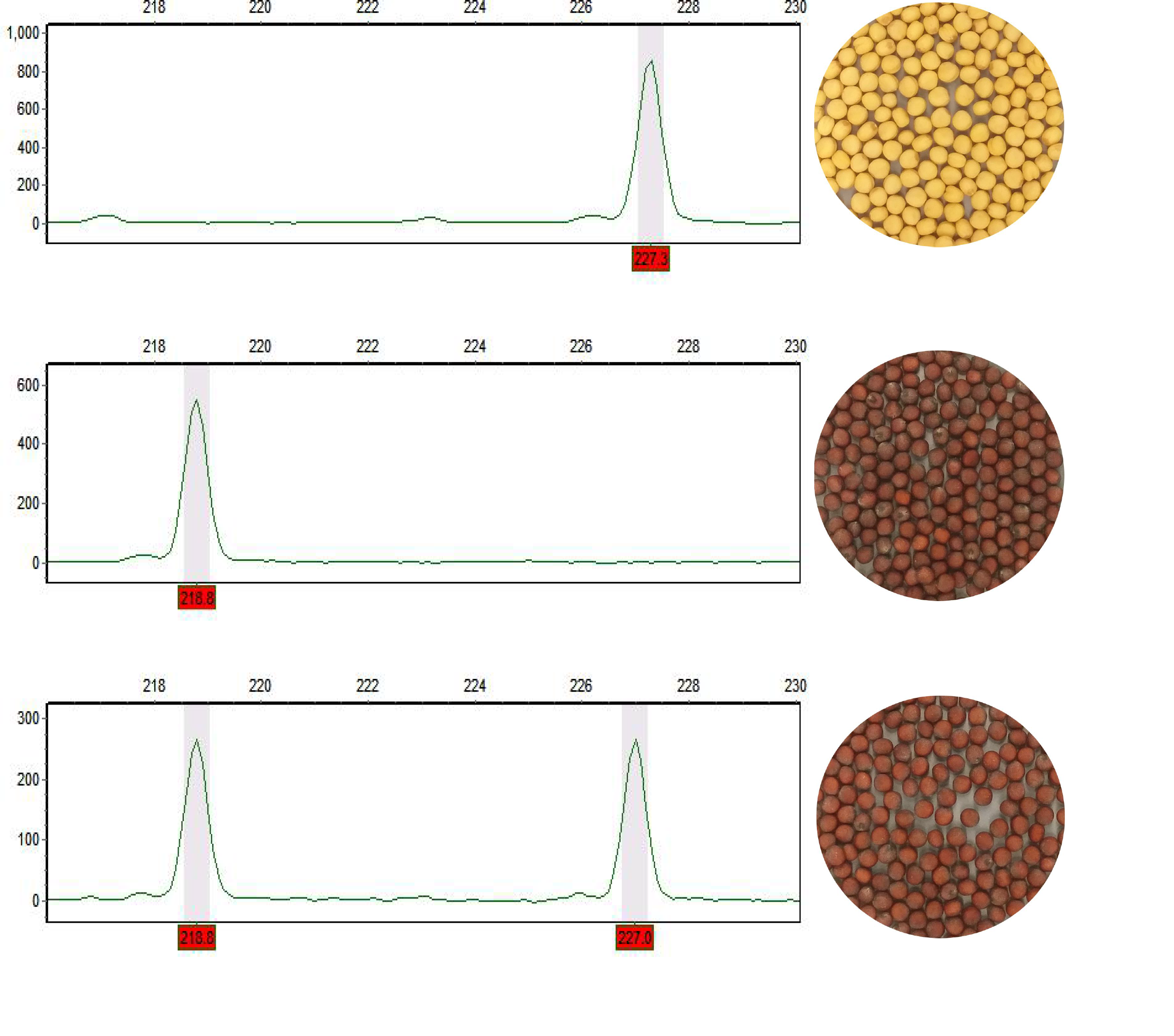

Supplement: S1 Fig — (TIF) [file pone.0209982.s001.tif]

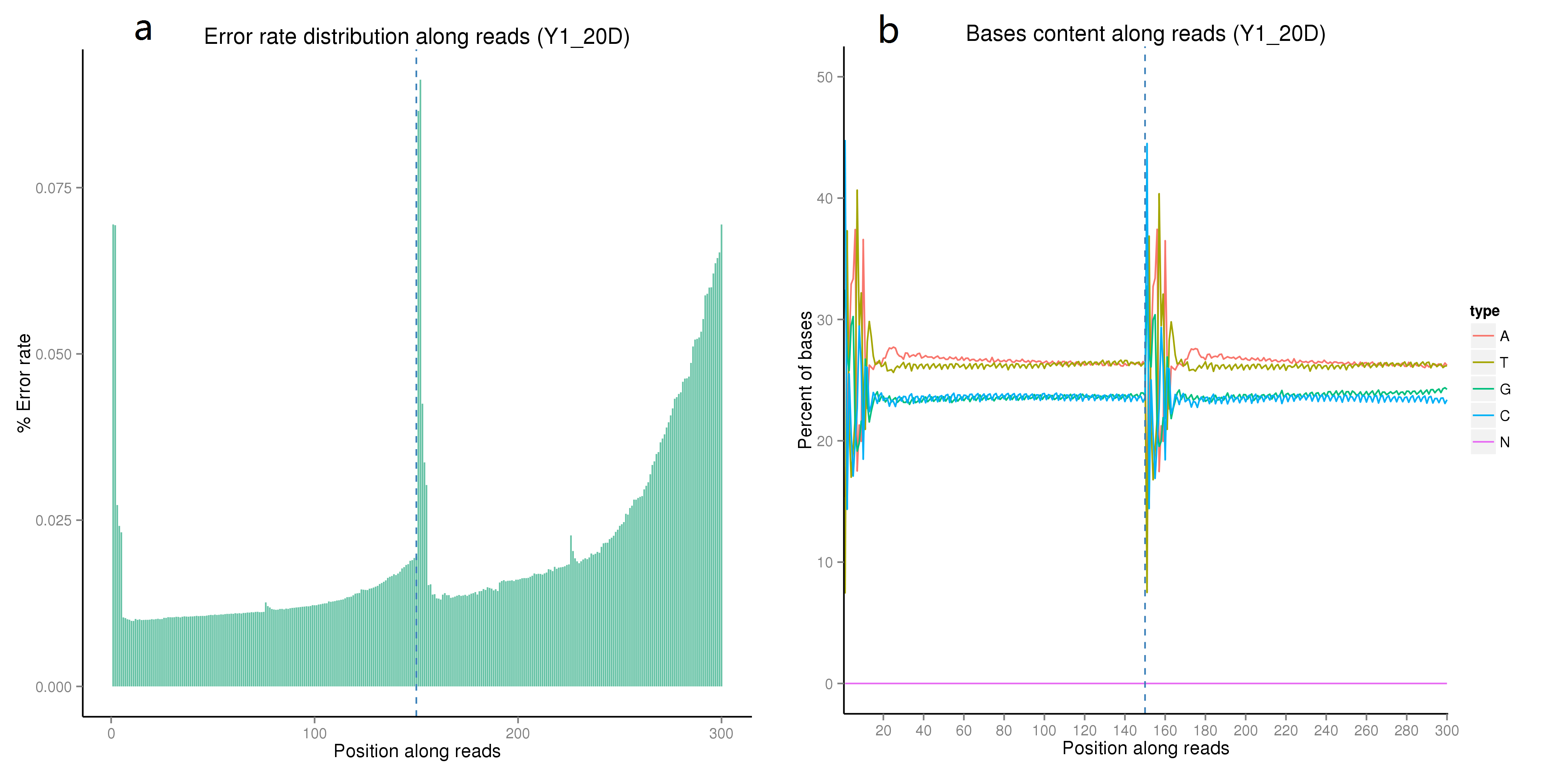

Supplement: S2 Fig — Error rates (a) and GC content (b) distribution of the transcriptome sequencing reads in yellow seed sample (Y1) of BC4S1 at 20 DAP. (TIF) [file pone.0209982.s002.tif]

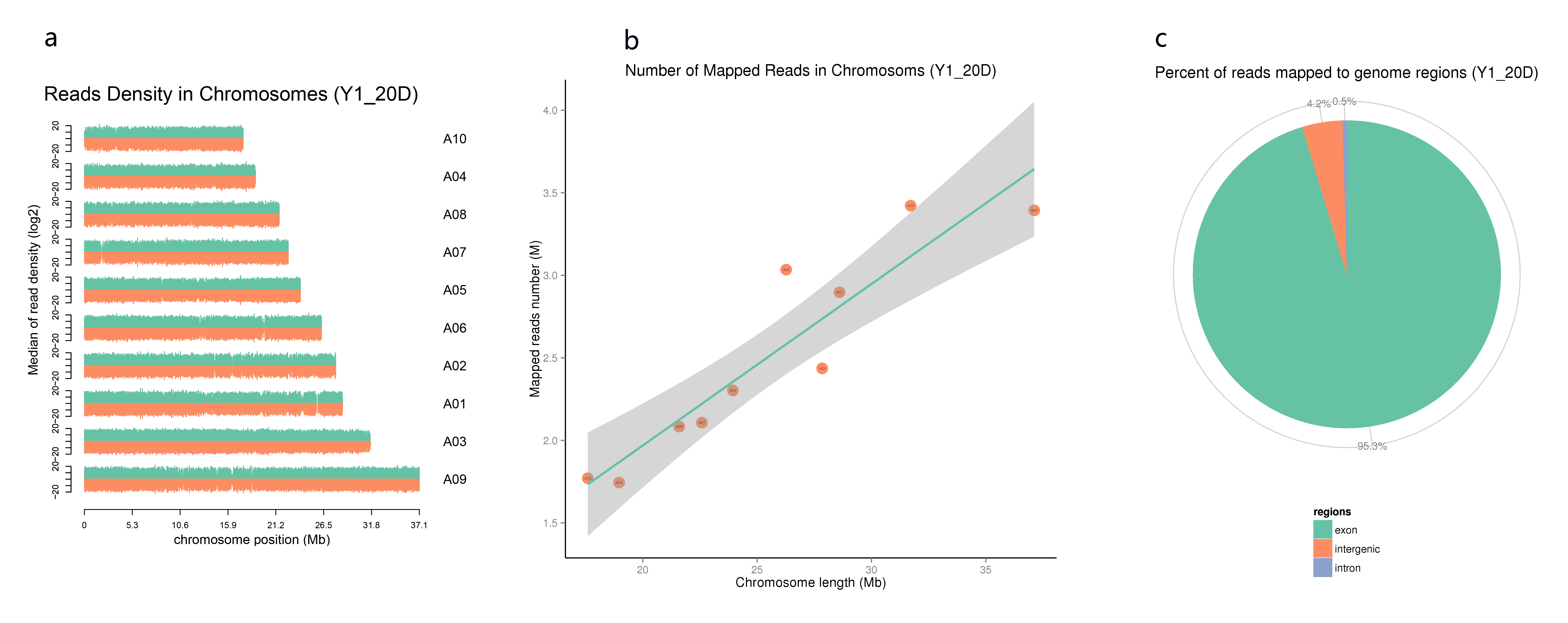

Supplement: S3 Fig — (TIF) [file pone.0209982.s003.tif]

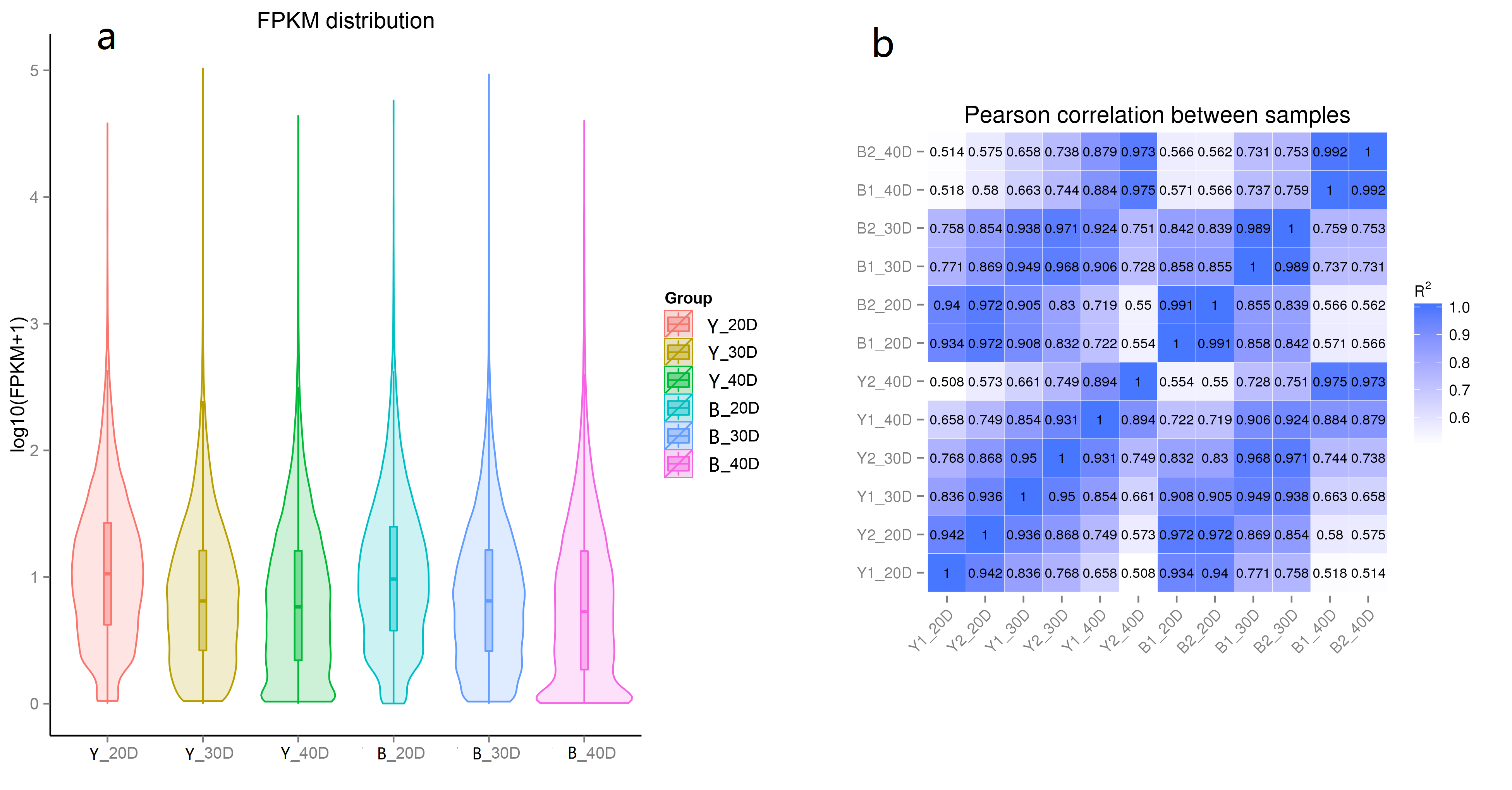

Supplement: S4 Fig — (TIF) [file pone.0209982.s004.tif]

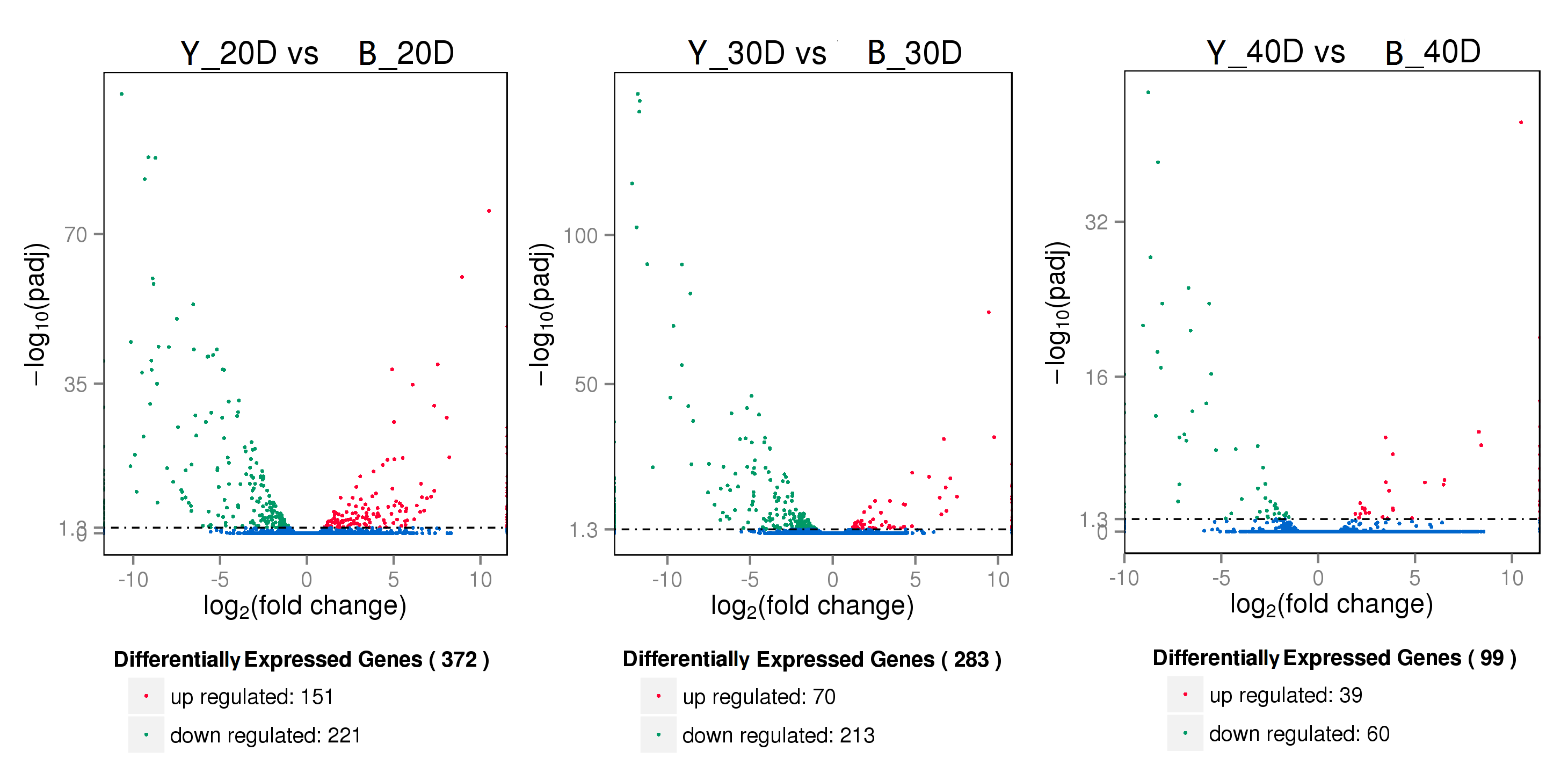

Supplement: S5 Fig — (padj value>1.3). (TIF) [file pone.0209982.s005.tif]

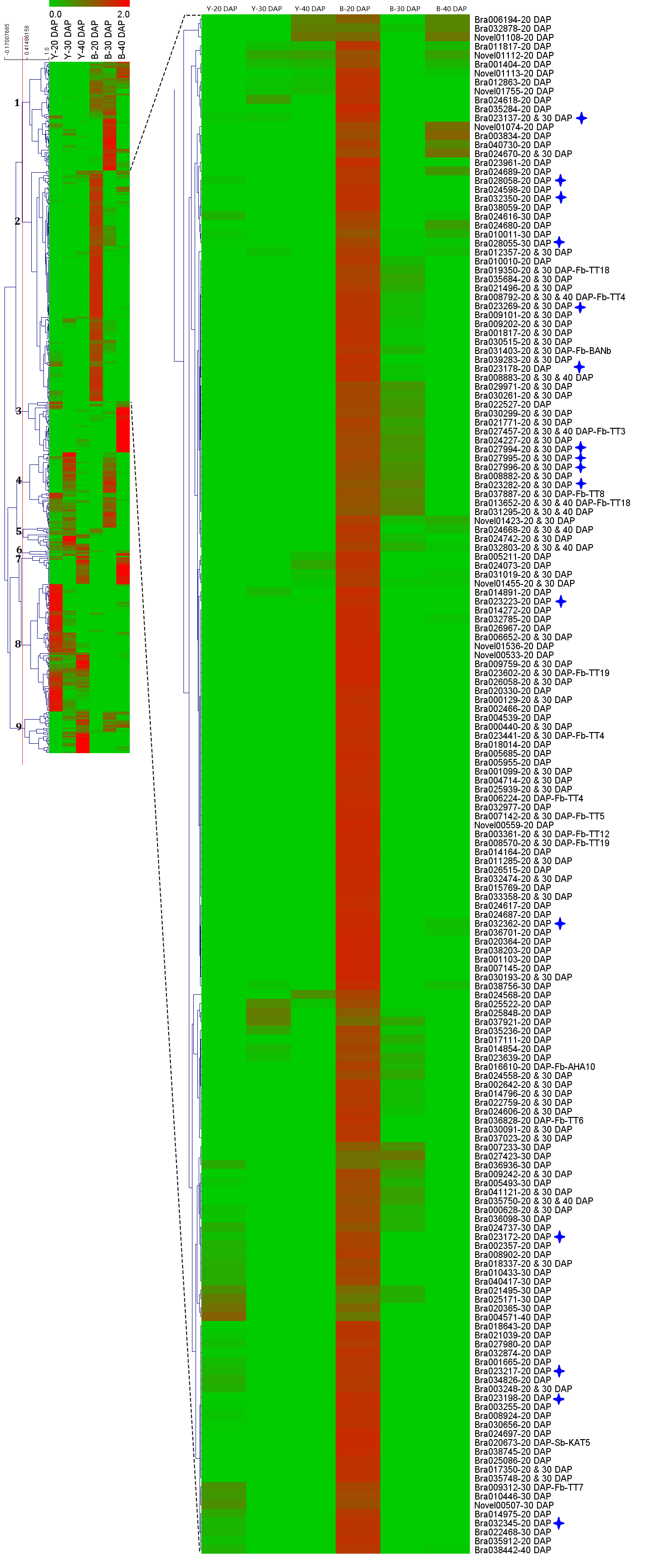

Supplement: S6 Fig — The sub-cluster 2 is enlarged; Fb = flavonoid biosynthesis pathway, Sb = subrine biosynthesis pathway. The DEGs from the major QTL SCA9-2 region of A9 chromosome are labelled with blue asterisks. (TIF) [file pone.0209982.s006.tif]

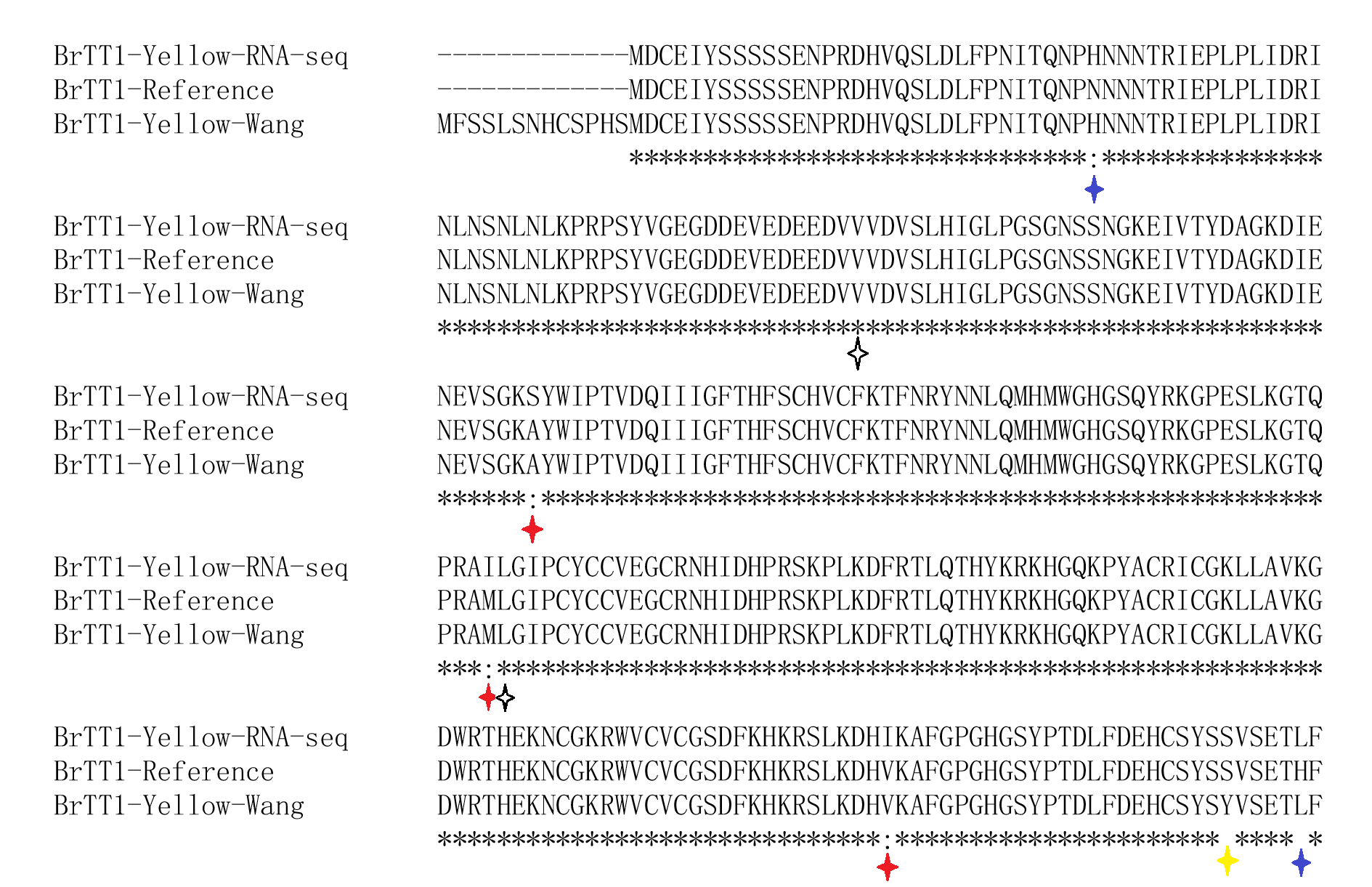

Supplement: S7 Fig — The two silent mutations, two sense mutations which were common in our materials and in Dahuang, one sense mutation found only in Dahuang, and three novel sense mutations found in our materials are marked with blank, blue, yellow and red asterisks, respectively. (TIF) [file pone.0209982.s007.tif]

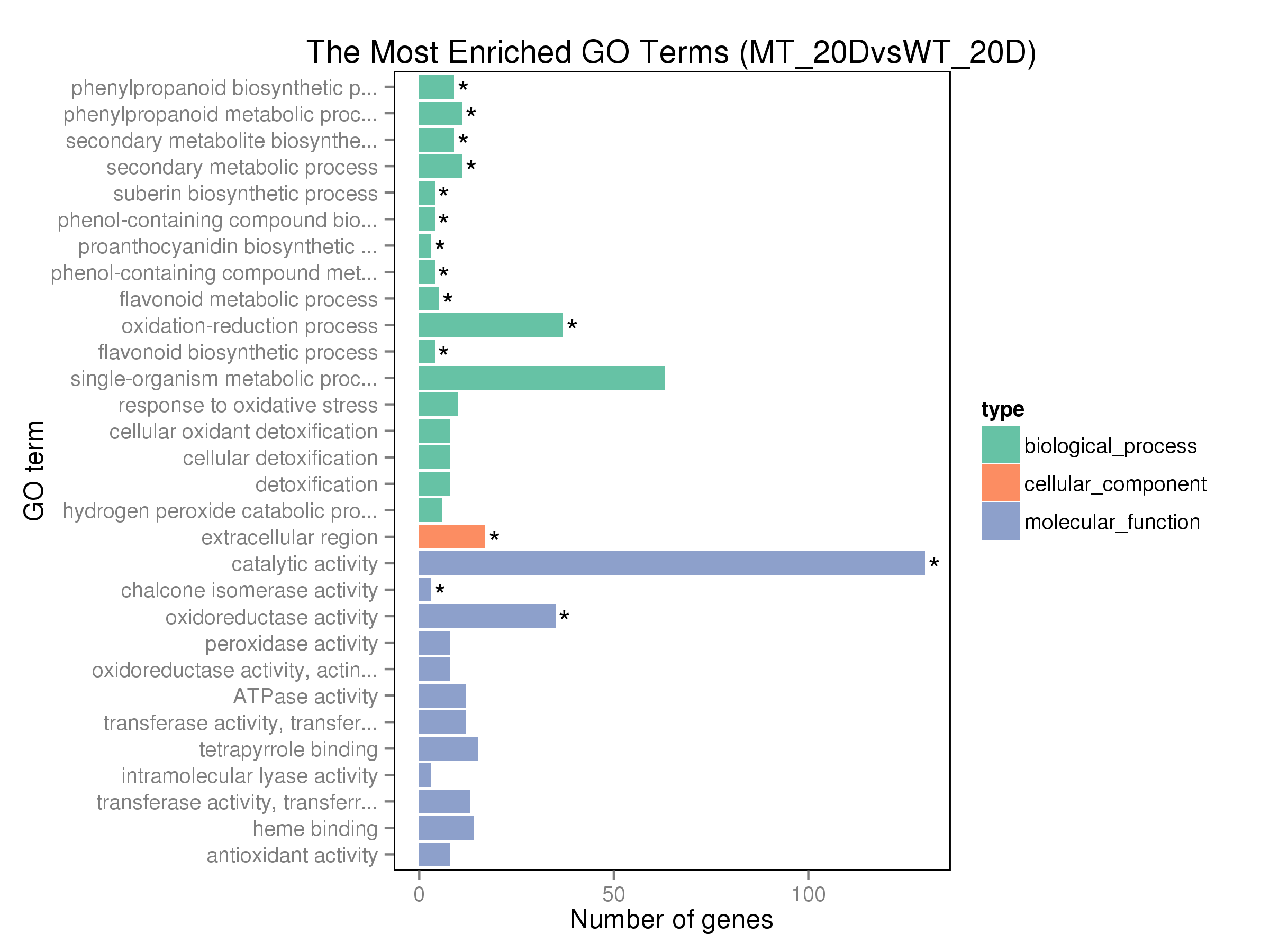

Supplement: S8 Fig — “*”indicates significantly enriched term. (TIF) [file pone.0209982.s008.tif]

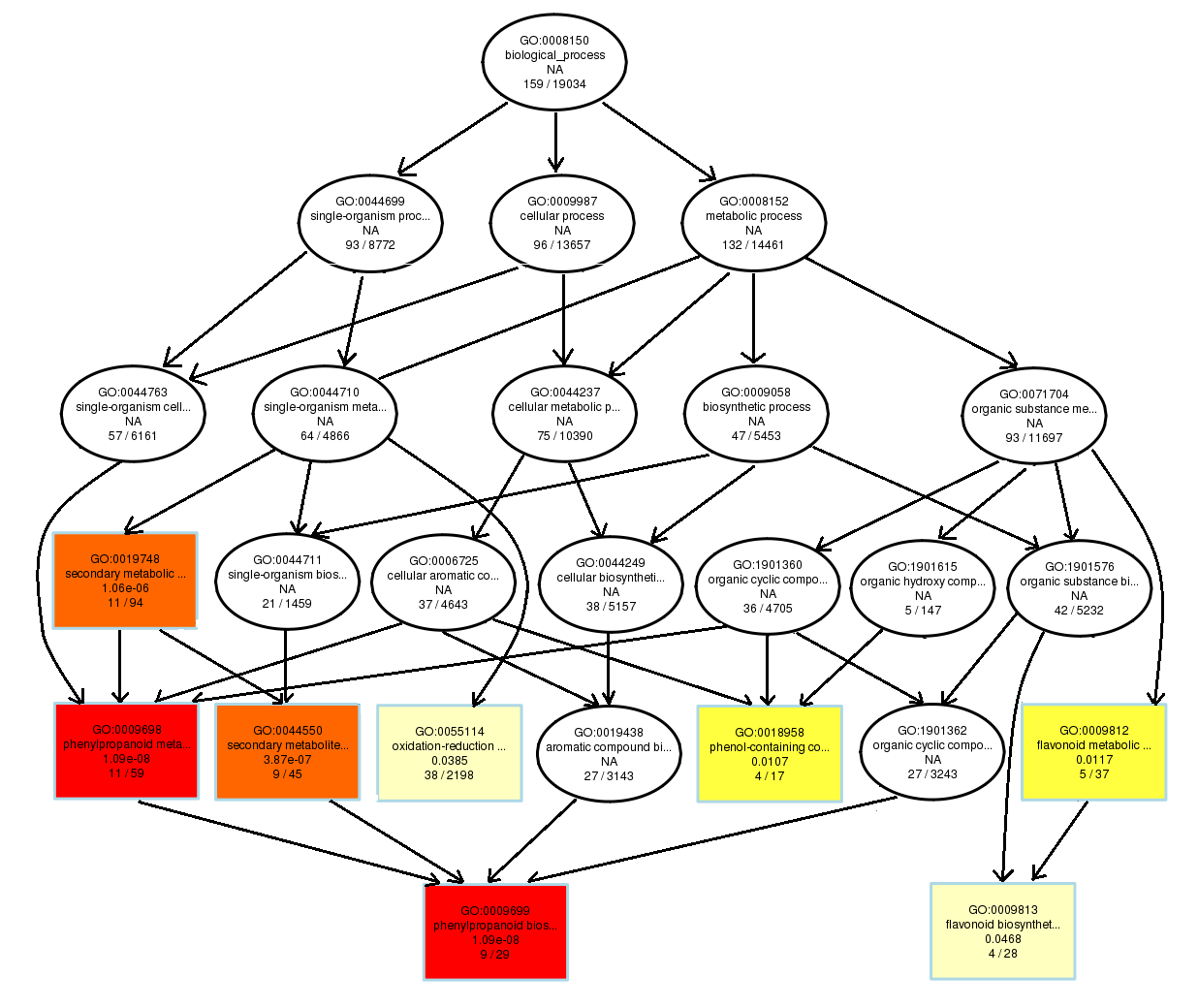

Supplement: S9 Fig — The top eight enriched GO terms (corrected p-value <0.05) are shown in square box and the related GO terms are shown in circles. The enrichment degree is illustrated by color shades where the red and yellow shades indicate the higher and lower enrichment degrees respectively. GO term’s name, description, corrected p-value, and the number of DEGs/background genes is listed in the boxes. (TIF) [file pone.0209982.s009.tif]

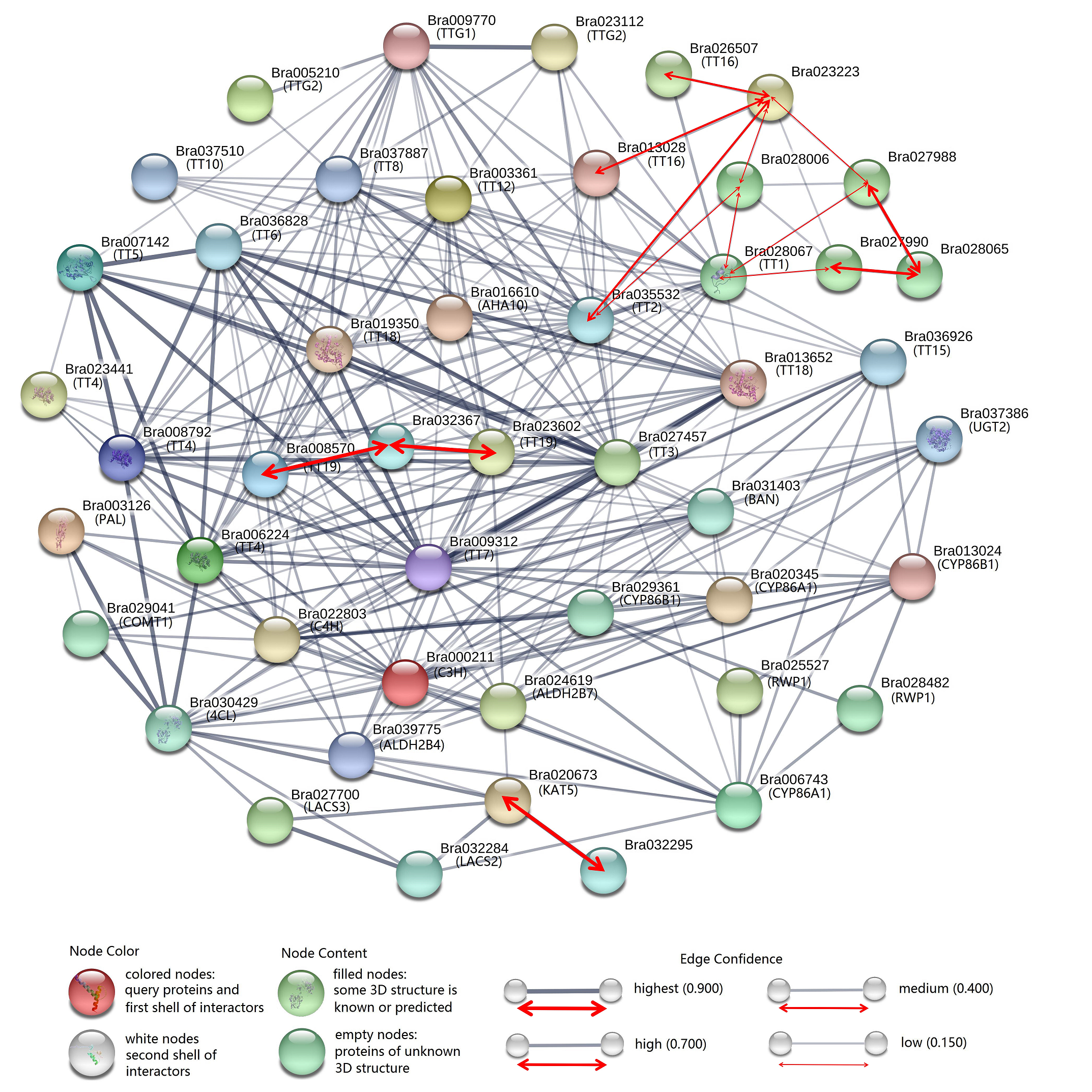

Supplement: S10 Fig — The names of the genes are given in brackets except for the seven genes from the SCA9-2 QTL region. Interaction of the DEGs from the QTL region and the different pathway genes are indicated by red arrows. Edge confidence: The thickness of the line indicates the strength of the supporting data, including neighborhood on chromosome, gene fusion, phylogenetic co-occurrence, homology, co-expression, experimentally determined interaction, database annotated, automated text mining, etc. (TIF) [file pone.0209982.s010.tif]

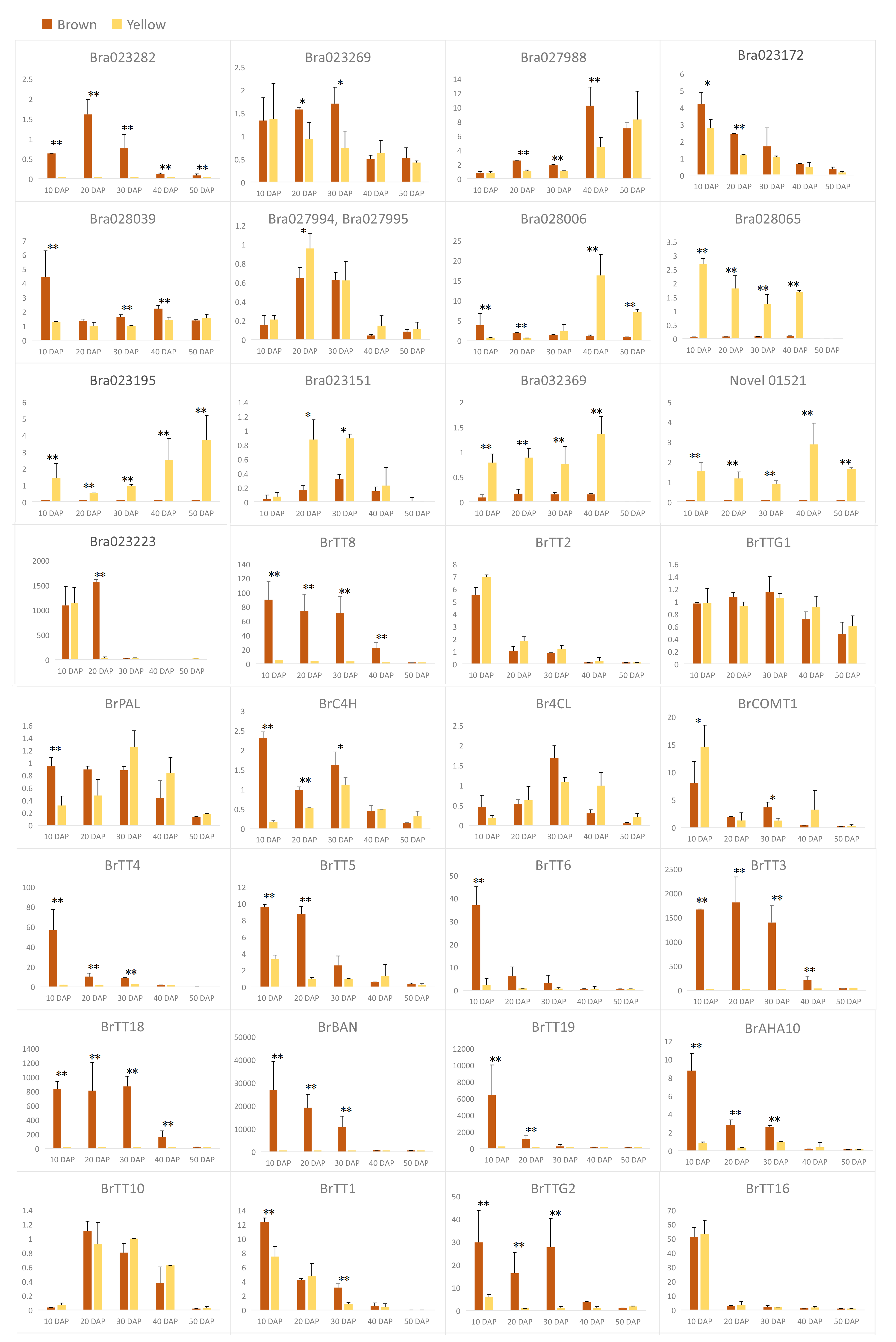

Supplement: S11 Fig — “*” = significant difference at 0.05 level (two-tailed); “**” = significant difference at 0.01 level (two-tailed). (TIF) [file pone.0209982.s011.tif]
